# Do helminth infections underpin urban‐rural differences in risk factors for allergy‐related outcomes?

**DOI:** 10.1111/cea.13335

**Published:** 2019-01-25

**Authors:** Gyaviira Nkurunungi, Lawrence Lubyayi, Serge A. Versteeg, Richard E. Sanya, Jacent Nassuuna, Joyce Kabagenyi, Prossy N. Kabuubi, Josephine Tumusiime, Christopher Zziwa, Robert Kizindo, Emmanuel Niwagaba, Carol Nanyunja, Margaret Nampijja, Harriet Mpairwe, Maria Yazdanbakhsh, Ronald van Ree, Emily L. Webb, Alison M. Elliott

**Affiliations:** ^1^ Immunomodulation and Vaccines Programme (MRC/UVRI and LSHTM) Uganda Research Unit Medical Research Council/Uganda Virus Research Institute Entebbe Uganda; ^2^ Department of Clinical Research London School of Hygiene and Tropical Medicine London UK; ^3^ Department of Epidemiology and Biostatistics School of Public Health University of the Witwatersrand Johannesburg South Africa; ^4^ Departments of Experimental Immunology and of Otorhinolaryngology Amsterdam University Medical Centers Amsterdam The Netherlands; ^5^ College of Health Sciences Makerere University Kampala Uganda; ^6^ Department of Parasitology Leiden University Medical Center Leiden The Netherlands; ^7^ MRC Tropical Epidemiology Group Department of Infectious Disease Epidemiology London School of Hygiene and Tropical Medicine London UK

**Keywords:** allergy, effect modification, helminths, risk factors, Uganda, urban‐rural

## Abstract

**Background:**

It is proposed that helminth exposure protects against allergy‐related disease, by mechanisms that include disconnecting risk factors (such as atopy) from effector responses.

**Objective:**

We aimed to assess how helminth exposure influences rural‐urban differences in risk factors for allergy‐related outcomes in tropical low‐ and middle‐income countries.

**Methods:**

In cross‐sectional surveys in Ugandan rural *Schistosoma mansoni* (*Sm*)‐endemic islands, and in nearby mainland urban communities with lower helminth exposure, we assessed risk factors for atopy (allergen‐specific skin prick test [SPT] reactivity and IgE [asIgE] sensitization) and clinical allergy‐related outcomes (wheeze, urticaria, rhinitis and visible flexural dermatitis), and effect modification by *Sm* exposure.

**Results:**

Dermatitis and SPT reactivity were more prevalent among urban participants, urticaria and asIgE sensitization among rural participants. Pairwise associations between clinical outcomes, and between atopy and clinical outcomes, were stronger in the urban survey. In the rural survey, SPT positivity was inversely associated with bathing in lakewater, *Schistosoma‐*specific IgG4 and *Sm* infection. In the urban survey, SPT positivity was positively associated with age, non‐Ugandan maternal tribe, being born in a city/town, BCG scar and light *Sm* infection. Setting (rural vs urban) was an effect modifier for risk factors including *Sm‐* and *Schistosoma‐*specific IgG4. In both surveys, the dominant risk factors for asIgE sensitization were *Schistosoma*‐specific antibody levels and helminth infections. Handwashing and recent malaria treatment reduced odds of asIgE sensitization among rural but not urban participants. Risk factors for clinical outcomes also differed by setting. Despite suggestive trends, we did not find sufficient evidence to conclude that helminth (*Sm*) exposure explained rural‐urban differences in risk factors.

**Conclusions and clinical relevance:**

Risk factors for allergy‐related outcomes differ between rural and urban communities in Uganda but helminth exposure is unlikely to be the sole mechanism of the observed effect modification between the two settings. Other environmental exposures may contribute significantly.

## 1 INTRODUCTION

Advances in health and hygiene practices have transformed high‐income countries into “cleaner” environments, with reduced infection exposure. Consequently, homeostatic immunomodulatory effects of exposure to microbes and parasites that co‐evolved with mammalian species (the “old friends hypothesis”) have been lost.^1^ The surge in allergy‐related diseases alongside other chronic inflammatory diseases in high‐income countries over recent decades has been partly attributed to this phenomenon.^2^ Although other environmental exposures^3^ may contribute, substantial support for the “old friends hypothesis” comes from studies in high‐income countries,^4^, ^5^, ^6^, ^7^, ^8^, ^9^ which show that traditional farming and related microbial exposures^10^ are associated with protection against allergy‐related diseases. Additional evidence suggests a parallel relationship between ongoing urbanization and increasing allergy‐related disease prevalence in tropical low‐ and middle‐income countries (LMICs).^11^, ^12^

Akin to farming environments in high‐income countries, rural LMIC settings are relatively protected against allergy‐related diseases.^13^, ^14^, ^15^, ^16^, ^17^ Animal models and in vitro experiments in human samples have identified helminths as potent inhibitors of allergic reactions,^18^, ^19^, ^20^ leading to the hypothesis that they are partly responsible for the low overall prevalence of allergy‐related diseases in tropical LMICs and the observed rural‐urban disparities in allergy‐related disease prevalence in the same settings.^16^, ^21^ Helminths may dissociate risk factors, such as atopy, from allergy‐related disease: work in Ugandan children showing that hookworm infection dissociates allergen‐specific IgE from the effector phase of the allergic response^22^ is strongly suggestive. However, little comparative analysis of risk factors for allergy in rural vs urban LMIC settings has been conducted. Exploration of these factors in LMICs, where an epidemiological transition is ongoing, provides an unprecedented opportunity to better understand interactions between the environment and the allergic pathway and allergy‐related disease outcomes.

Using data generated from two surveys in Uganda, one in rural helminth‐endemic Lake Victoria island fishing villages and another in nearby mainland urban communities with lower helminth exposure, we investigated socio‐demographic, behavioural, clinical and immunological characteristics as risk factors for allergy‐related outcomes and assessed whether helminth infections contribute to rural‐urban differences in these risk factors.

## 2 METHODS

### 2.1 Study settings and procedures

Rural participants were residents of 26 helminth‐endemic fishing villages of Koome islands, Mukono district, Uganda (population 18 778 in 2014^23^). Urban participants were residents of Entebbe Municipality, a lower helminth exposure area situated on the northern shores of Lake Victoria, 40 km southwest of the Ugandan capital, Kampala, and 35 km from Koome. The municipality had approximately 69 430 inhabitants in 2014,^23^ distributed across 24 sub‐wards, the smallest administrative units.

The “rural survey” was part of the Lake Victoria Island Intervention Study on Worms and Allergy‐related diseases (LaVIISWA; ISRCTN47196031), a cluster‐randomized trial of standard vs intensive anthelminthic intervention, described elsewhere.^24^, ^25^ A baseline household survey preceded the trial intervention; helminth‐allergy associations at baseline have been reported.^24^ A household‐based allergy outcomes survey (the “rural survey”) was conducted between September 2015 and August 2016, following 3 years of anthelminthic intervention: there was no difference in the prevalence of allergy outcomes between the two trial arms.^26^ Sampling for the survey involved random selection of 70 households from each village using a Stata program. All household members (1 year and older) of selected households were then invited to participate. Permission for household participation was granted by the household head.

The urban survey of allergy‐related outcomes (September 2016–September 2017) was designed intentionally to collect data from Entebbe municipality for comparison with the helminth‐endemic rural survey. Before the start of the survey, each sub‐ward was mapped onto satellite imagery of the municipality. A random point generation function of ArcGIS software (version 10.4.1, Environmental Systems Research Institute, Redlands, CA) was then used to generate random starting points within each sub‐ward. The number of starting points selected was proportional to the population size of the sub‐ward. Coordinates of the random starting points generated were loaded onto geographic information system (GIS) devices (eTrex®, Garmin™ Ltd, Olathe, KS). These devices were then used in the field to identify the selected random points, from which the nearest four houses were surveyed.

There was no randomization to intensive or standard anthelminthic treatment in the urban survey; however, all other procedures were designed to be identical in both the urban and the rural survey.

Following written informed consent and assent, questionnaires were completed for each participant, capturing socio‐demographic, clinical and behavioural characteristics as well as asthma, eczema and allergy symptoms. The latter employed questions based on the International Study on Allergy and Asthma in Children (ISAAC) questionnaire. Blood, stool and mid‐stream urine were collected. Blood samples were used for haemo‐parasitology, HIV serology and storage of plasma and cells for immunoassays. One stool sample per participant was examined for intestinal helminth infections using the Kato‐Katz method^27^ (two slides, read by different technologists). The remaining sample was stored and later investigated for *Schistosoma mansoni (Sm), Strongyloides stercoralis* and hookworm (*Necator americanus*) infections using multiplex real‐time PCR.^28^, ^29^ Urine was assessed for *Sm* circulating cathodic antigen (CCA, Rapid Medical Diagnostics, Pretoria, South Africa). *Schistosoma* egg [SEA]‐ and adult worm [SWA] antigen‐specific immunoglobulin (Ig)E, IgG4 and IgG levels were assessed in plasma using in‐house ELISAs (Data S1).

Ethics committees of Uganda Virus Research Institute (refs: GC/127/12/05/03 and GC/127/16/02/547) and London School of Hygiene and Tropical Medicine, (refs: 6187 and 10709) and the Uganda National Council for Science and Technology (ref: HS1183 and HS2036) approved both surveys.

### 2.2 Allergy‐related outcomes

Outcomes were skin prick test (SPT) reactivity to allergens common in our setting,^30^ allergen‐specific IgE (asIgE) sensitization, self‐reported recent (previous 12 months) wheeze, recent rhinitis, recent urticarial rash and visible flexural dermatitis.

Skin prick test reactivity (wheal ≥3 mm diameter after 15 minutes in the presence of saline [negative] and histamine [positive] controls) to dust mites (*Dermatophagoides* mix, *Blomia tropicalis*) and German cockroach (*Blattella germanica*) (ALK‐Abelló; supplied by Laboratory Specialities [Pty] Ltd., Randburg, South Africa) was assessed using standard procedures.^31^ SPT reactivity was defined primarily as a positive response to any of the three allergens. SPT reactivity was also analysed as a positive vs negative response to individual allergens.

Whole allergen (*Dermatophagoides pteronyssinus*, peanut [*A hypogaea]* and *B germanica*) extract‐specific plasma IgE (asIgE) was measured by ImmunoCAP® (ThermoFisher Scientific, Uppsala, Sweden) in a sample of 780 and 345 rural and urban survey participants, respectively, randomly selected from those with sufficient volume of stored plasma. Allergen‐specific IgE sensitization was defined as a positive ImmunoCAP response (IgE concentration ≥0.35kU/L) to any of the three allergens and as a positive vs negative ImmunoCAP response for individual allergens. ImmunoCAP IgE outcomes were also analysed as continuous variables.

Wheeze is considered a good proxy for asthma in epidemiological studies^32^ and was assessed separately in two age groups (≥5 years and <5 years) using an interviewer‐administered ISAAC questionnaire. The principal age group of interest was ≥5 years because wheeze cannot be assumed to represent asthma in children below 5 years.^33^

Data on recent rhinitis (runny/blocked nose or sneezing accompanied by watery and itchy eyes, in the absence of cold or “flu”) and urticarial rash (pruritic rash with weals, known as “ebilogologo” in the local language [Luganda]) were obtained by questionnaire. Visible flexural dermatitis was assessed (by staff trained on Williams’ online manual^34^) as an erythematous rash with surface change in and around skin creases.^35^, ^36^

### 2.3 Statistical methods

Data analysis was conducted using Stata 13.1 (College Station, TX). The following were assessed as potential risk factors for allergy‐related outcomes: socio‐demographic characteristics (age, sex, presence of older/younger siblings, maternal tribe, paternal tribe, location of birth and occupation), behavioural characteristics (frequency of lake contact, type of bathing water, handwashing behaviour, footwear outside the house, smoking and alcohol use), clinical characteristics (helminth infections, exposure to anthelminthic treatment in utero, anthelminthic treatment in last 12 months, parental history of allergies, BCG scar, immunisation history, malaria treatment in last 12 months, malaria infection and HIV infection) and immunological characteristics (plasma SEA‐ and SWA‐specific IgE, IgG4 and IgG levels). Additionally, allergy‐related outcomes were independently assessed as risk factors for each other.

Stata “svy” commands were used to allow for clustering of participants within villages and for the non‐self‐weighting design of the rural survey^24^ and for clustering by sub‐ward in the urban survey.

Logistic regression was used to compare the prevalence of outcomes and other characteristics between the rural and urban survey and to assess associations between each pair of allergy‐related outcomes in both surveys. Population attributable fractions (PAFs) for pairs of allergy‐related outcomes were calculated. Interaction tests were done to assess whether these associations differed by setting. Unadjusted and adjusted odds ratios (OR) for associations between exposures and allergy‐related outcomes were estimated using univariable and multivariable logistic regression. Additionally, linear regression was used in secondary analyses of ImmunoCAP IgE outcomes as continuous variables. Age, sex (a priori) and factors showing evidence of crude association with an outcome (*P* < 0.05) were considered in multivariable analyses for that outcome. We hypothesized that helminth infections might be key mediating factors on the causal pathway between urban/rural residence and allergy‐related outcomes; hence, helminths (and *Sm‐*specific antibody responses and other “helminth‐related” factors such as frequency of lake contact and occupation) were not included in multivariable analyses for other risk factors. The potential mediating role of helminths was then investigated separately by assessing whether associations between non‐helminth‐related risk factors and allergy‐related outcomes changed substantially when adjusted for *Sm* infections and *Schistosoma*‐specific antibody levels. These analyses were initially conducted separately for each survey. Subsequently, we merged data from the two surveys and tested for interaction between the rural and urban survey, to assess whether risk factors for allergy outcomes differed by setting. Here, we also assessed the potential role of helminths in urban‐rural interactions by comparing interaction *P* values before and after adjusting for *Sm* infection. A 5% significance level was used for all analyses.

## 3 RESULTS

### 3.1 Participants’ characteristics

**Figure 1:**
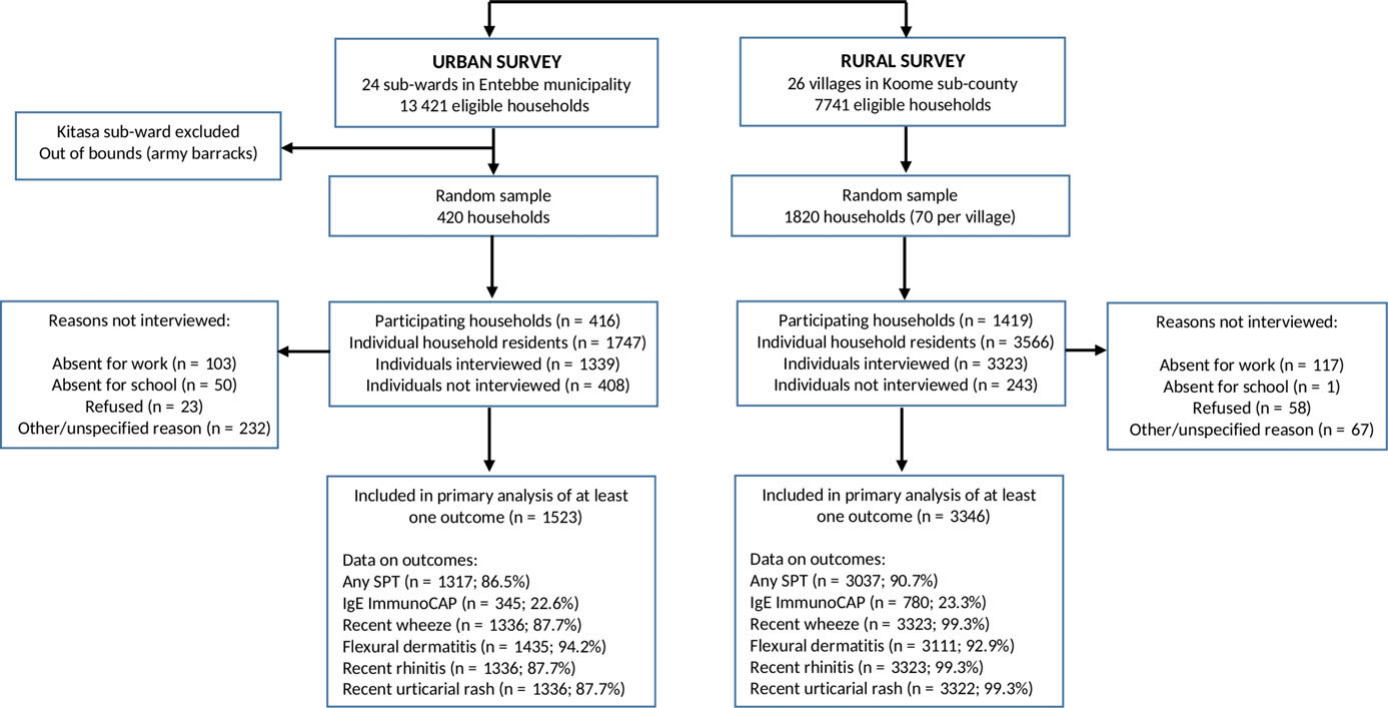
Study flowchart

Flowcharts of the surveys are shown in Figure 1. Of 1820 households randomly selected for the rural survey (70 from each of the 26 villages), 1419 (78%) took part. There were 3566 individuals inhabiting the 1419 participating households; 3323 (93.2%) were interviewed and 3346 (93.8%) had data on at least one allergy‐related outcome. Of 420 households randomly selected for the urban survey, 416 (99%) took part. There were 1747 individuals inhabiting the 416 households; 1339 (77%) were interviewed and 1523 (87%) had data on at least one allergy‐related outcome.

**Figure 2:**
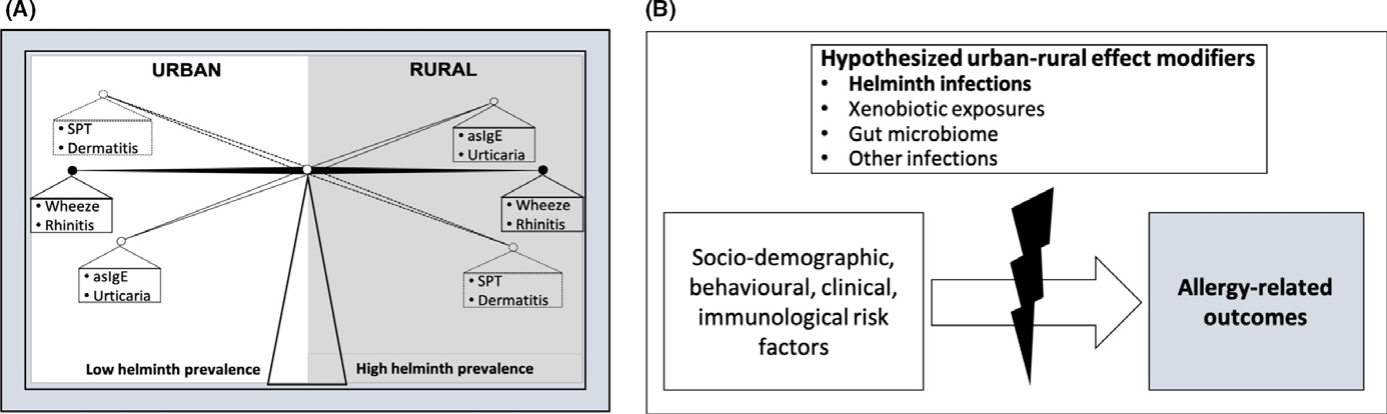
Urban‐rural differences in risk factors for allergy‐related outcomes in Uganda: a role for helminths? A, summary of principal findings regarding prevalence of allergy‐related outcomes in urban Uganda and in rural Ugandan fishing communities. B, Risk factors for allergy‐related outcomes differed between urban and rural settings. Our data suggest that helminth exposure is unlikely to be the only factor involved in this effect modification. Additional hypothesized effect modifiers are indicated

Participant characteristics differed between the two study settings (Table 1). Significantly, rural, compared to urban participants, were more likely to be infected with helminths (including *Sm*), malaria and HIV, had higher median levels of *Schistosoma*‐specific antibodies and were more likely to report anthelminthic or malaria treatment in the previous 12 months. Dermatitis and SPT reactivity were more prevalent among urban participants, while asIgE sensitization and urticaria were more common among rural participants (Table 1 and Figure 2A). The prevalence of wheeze and rhinitis was similar between the two communities.

**Table 1:** Characteristics of study participants

Characteristics	Urban survey n/N (%)^a^	Rural survey n/N (%)^a^	*P* value^b^
Socio‐demographic			
Age in (y), median (IQR)	20 (8, 31)	24 (8, 34)	0.329^c^
Male sex	688/1610 (42.7)	**1738/3350 (49.5)**	0.002
Place of birth			
City	**53/513 (10.3)**	61/2406 (2.9)	
Town	**138/513 (26.9)**	254/2406 (10.4)	
Village	322/513 (62.7)	**2091/2406 (86.7)**	<0.001
Maternal tribe, larger region grouping			
Central Uganda	605/1331 (45.5)	1197/3304 (36.5)	
Other, Ugandan	607/1331 (45.6)	1588/3304 (48.1)	
Non‐Ugandan, African	119/1331 (8.9)	519/3304 (15.4)	0.020
Paternal tribe, larger region grouping			
Central Uganda	593/1334 (44.5)	1343/3317 (39.8)	
Other, Ugandan	624/1334 (46.8)	1556/3317 (47.5)	
Non‐Ugandan, African	117/1334 (8.7)	418/3317 (12.7)	0.208
Maternal history of allergies (general)	93/1187 (7.8)	**366/2930 (12.7)**	<0.001
Paternal history of allergies (general)	30/1117 (2.6)	**171/2796 (5.7)**	0.005
Maternal history of asthma	27/1266 (2.1)	93/2931 (3.4)	0.167
Paternal history of asthma	27/1218 (2.2)	62/2796 (2.3)	0.950
Maternal history of eczema	35/1229 (2.8)	131/2931 (4.5)	0.206
Paternal history of eczema	15/1159 (1.3)	**96/2795 (2.9)**	0.028
Occupation, grouped by type			
Student or child (not at school)	**662/1338 (49.5)**	1166/3323 (36.7)	
Unemployed or housewife	**292/1338 (21.8)**	301/3323 (8.7)	
Agricultural, fishing or lake related	60/1338 (4.5)	**1389/3323 (38.8)**	
Professional or service providers (Shops, saloons, bars, restaurants, entertainment)	**324/1338 (24.2)**	467/3323 (15.6)	<0.001
Helminth infections			
*S mansoni* (KK)	86/1197 (7.2)	**846/2751 (31.8)**	<0.001
*S mansoni* intensity (KK)			
Uninfected	**1111/1197 (92.8)**	1905/2751 (68.2)	
Low	41/1197 (3.4)	**425/2751 (15.7)**	
Moderate	31/1197 (2.6)	**231/2751 (9.1)**	
Heavy	14/1197 (1.1)	**190/2751 (7.1)**	<0.001
* S mansoni* (urine CCA)	581/1318 (44.1)	**2445/2879 (85.6)**	<0.001
* S mansoni* (PCR)	204/1191 (17.1)	**1338/2747 (50.0)**	<0.001
* A lumbricoides* (KK)	0/1197 (0.0)	14/2751 (0.4)	
* Trichuris trichiura* (KK)	21/1196 (1.8)	**245/2751 (7.8)**	<0.001
* N americanus* (PCR)	56/1191 (4.7)	**259/2747 (8.4)**	0.016
* S stercoralis* (PCR)	29/1191 (2.4)	**190/2747 (6.2)**	<0.001
*Schistosoma‐*specific antibody levels			
* *SEA‐specific IgE (μg/mL), median (IQR)	2.7 (2.6, 2.8)	**4.6 (4.3, 4.8)**	<0.001^c^
* *SWA‐specific IgE (μg/mL), median (IQR)	2.2 (2.1, 2.4)	**4.9 (4.6, 5.1)**	<0.001^c^
* *SEA‐specific IgG4 (μg/mL), median (IQR)	30.8 (27.8, 37.3)	**278.6 (228.7, 322.4)**	<0.001^c^
* *SWA‐specific IgG4 (μg/mL), median (IQR)	42.7 (40.5, 44.1)	**108.6 (98.3, 124.7)**	<0.001^c^
* *SEA‐specific IgG (μg/mL), median (IQR)	777.9 (744.6, 806.1)	**1975.4 (1848.0, 2096.4)**	<0.001^c^
* *SWA‐specific IgG (μg/mL), median (IQR)	795.4 (771.2, 828.6)	**1497.2 (1429.4, 1561.5)**	<0.001^c^
Allergy‐related outcomes			
Skin prick test reactivity			
Any	**302/1317 (22.9)**	576/3037 (19.1)	0.054
*Dermatophagoides* mix	**228/1317 (17.3)**	326/3037 (10.5)	<0.001
*B tropicalis*	**184/1317 (13.9)**	229/3036 (7.9)	<0.001
*B germanica*	186/1320 (14.1)	350/3035 (11.8)	0.137
Allergen‐specific IgE (≥0.35 kU/L, ImmunoCAP)			
Any	148/345 (42.9)	**437/780 (55.1)**	0.007
*D pteronyssinus*	104/345 (30.1)	264/780 (33.2)	0.421
*B germanica*	118/345 (34.2)	**393/780 (49.8)**	<0.001
*A hypogaea*	41/345 (11.8)	114/780 (14.9)	0.266
Total IgE (kU/L), median (IQR)	159 (56, 522)	**672 (249, 1942)**	<0.001
Wheeze in last 12 mo, age<5 y	3/229 (1.3)	9/547 (1.4)	0.972
Wheeze in last 12 mo, age ≥ 5 y	24/1107 (2.2)	87/2776 (3.2)	0.190
Visible flexural dermatitis	**22/1435 (1.5)**	5/3111 (0.1)	<0.001
Rhinitis in last 12 mo	45/1336 (3.4)	104/3323 (3.2)	0.806
Urticarial rash in last 12 mo	53/1336 (3.9)	**334/3322 (9.9)**	<0.001
Other			
Any worm treatment in the last 12 mo	795/1296 (61.3)	**2938/3307 (87.7)**	<0.001
Malaria treatment in the last 12 mos	506/1336 (37.8)	**1993/3323 (60.8)**	<0.001
*P falciparum* positivity by blood smear	3/1347 (0.2)	**102/2923 (3.7)**	<0.001
HIV infection	66/1339 (4.9)	**402/2399 (17.3)**	<0.001

CCA: circulating cathodic antigen; IQR: interquartile range; KK: Kato‐Katz; PCR: polymerase chain reaction; SEA: Schistosoma egg antigen; SWA: Schistosoma adult worm antigen.

a Percentages adjusted for survey design. Percentages that are significantly higher in one setting compared to the other (*P *≤* *0.05) are highlighted in bold. Adjusting for age and sex differences had no significant impact on these differences.

b *P* values obtained from survey design‐based logistic regression.

c *P* values obtained from survey design‐based linear regression.

### 3.2 Associations between allergy‐related outcomes

Crude associations between allergy‐related outcomes are shown in Table 2. Individuals who were ImmunoCAP asIgE sensitized were more likely to have a positive SPT response in both surveys; the PAF for SPT reactivity associated with asIgE sensitization was 86.1% and 80.9% for the urban and rural survey, respectively. Atopy measures (asIgE, SPT) were generally more strongly associated with other allergy‐related conditions in the urban compared to rural survey; asIgE‐rhinitis (interaction *P* = 0.081), asIgE‐urticaria (interaction *P* = 0.056), SPT‐rhinitis (interaction *P* = 0.019) and SPT‐urticaria (interaction *P* = 0.005) associations approached statistical significance. Another major difference was that urticaria was associated with wheeze, rhinitis and SPT reactivity in the urban survey, but not with any allergy‐related outcome in the rural survey.

**Table 2:** Crude associations between allergy‐related outcomes

		SPT	Wheeze	Rhinitis	Urticaria
**asIgE**					
Urban	OR (95% CI)	**21.4 (10.2, 44.6)**	5.5 (0.4, 68.6)	**3.7 (1.2, 11.9)**	3.7 (0.8, 16.2)
	*P* value	**<0.001**	0.171	**0.028**	0.075
	PAF (95% CI)	86.1% (81.4, 88.3)	65.5% (−120, 78.8)	53.1% (12.1, 66.6)	53.1% (−18.2, 68.2)
Rural	OR (95% CI)	**10.3 (5.3, 19.8)**	**3.9 (1.3, 11.5)**	1.1 (0.5, 2.6)	0.9 (0.6, 1.3)
	*P* value	**<0.001**	**0.015**	0.793	0.651
	PAF (95% CI)	80.9% (72.7, 85.1)	62.2% (19.3, 76.5)	5.7% (−57.9, 35.6)	−4.7% (−35.8, 12.4)
	Interaction *P* value	0.127	0.792	0.081	**0.056**
**SPT**					
Urban	OR (95% CI)		2.2 (0.6, 8.1)	**6.5 (3.4, 12.5)**	**2.2 (1.6, 2.8)**
	*P* value		0.211	**<0.001**	**<0.001**
	PAF (95% CI)		23.4% (−28.6, 37.6)	54.2% (45.2, 58.9)	20.8% (14.4, 24.6)
Rural	OR (95% CI)		**3.0 (1.8, 5.1)**	**2.6 (1.7, 3.9)**	1.2 (0.9, 1.6)
	*P* value		**<0.001**	**<0.001**	0.243
	PAF (95% CI)		29.2% (17.6, 31.9)	23.4% (15.5, 27.9)	3.6% (−2.4, 20.9)
	Interaction *P* value		0.647	**0.019**	**0.005**
**Wheeze**					
Urban	OR (95% CI)			**7.4 (1.7, 33.2)**	**4.9 (1.1, 21.7)**
	*P* value			**0.011**	**0.035**
Rural	OR (95% CI)			**11.9 (5.7, 24.9)**	1.4 (0.6, 3.3)
	*P* value			**<0.001**	0.403
	Interaction *P* value			0.557	0.127
**Rhinitis**					
Urban	OR (95% CI)				**9.6 (5.6, 16.4)**
	*P* value				**<0.001**
Rural	OR (95% CI)				0.7 (0.3, 1.6)
	*P* value				0.429
	Interaction *P* value				**<0.001**

asIgE: ImmunoCAP IgE sensitization to any of *D pteronyssinus*,* A hypogaea*, or *B germanica* on ImmunoCAP; SPT: skin prick test reactivity to any of Dermatophagoides mix, *B tropicalis* or *B germanica*.

Odds ratios (ORs), *P* values and population attributable fractions (PAFs) were obtained from survey design‐adjusted analyses. Visible flexural dermatitis was not assessed because it was rare. Significant associations are highlighted in bold. Interaction *P* values are shown to denote whether tests for interaction showed statistical evidence for urban‐rural differences in associations between allergy‐related outcomes, or not.

We hypothesized that helminth infection, particularly *Sm* infection, might mediate this effect modification between the urban and rural setting (Figure 2B). However, the comparison of crude associations (reported above) with associations adjusted for current *Sm* infection (generally, or categorized by infection intensity) and *Schistosoma*‐specific antibody concentrations did not show clear differences in the test statistics (Table S1); hence, any mediating role of current *Sm* infection, including effects on interactions between the rural and urban survey, was not evident.

### 3.3 Factors associated with skin prick test reactivity

Table 3 and Table S2 show factors associated with SPT reactivity to any of *Dermatophagoides* mix, *B tropicalis* or *B germanica*. In the urban survey, increasing age, non‐Ugandan maternal tribe, being born in a city (compared to town or village) and having a BCG scar were positively associated with SPT reactivity. Additionally, light *Sm* infection (KK) and *Sm* infection (PCR) were positively associated with SPT reactivity in the urban survey, in sharp contrast to observations in the rural survey, where current *Sm* infection (KK, PCR and CCA) was associated with reduced odds of SPT reactivity. This rural‐urban difference was statistically significant (interaction *P* values = 0.002 and 0.015 for *Sm*‐PCR and *Sm*‐KK intensity, respectively). Other factors inversely associated with SPT reactivity in the rural survey were related to helminth infections and included bathing in lakewater and SWA‐specific IgG4.

**Table 3:** Factors associated with SPT reactivity to any of *Dermatophagoides* mix, *B tropicalis* or *B germanica*

Factor	Urban			Rural			Interaction *P*
	N (%)^a^	aOR (95% CI)^b^, ^c^	*P*	N (%)^a^	aOR (95% CI)^b^, ^d^	*P*	
Age		**1.02 (1.00, 1.03)**	**0.035**		**1.02 (1.00, 1.03)**	**0.015**	0.384
Sex							
Male	132 (26)	1		285 (18)	1		
Female	170 (21)	0.71 (0.49, 1.02)	0.061	291 (20)	1.09 (0.79, 1.52)	0.558	**0.015**
Older siblings (Yes/No)							
No	73 (22)	1		113 (24)	1		
Yes	194 (23)	1.58 (0.90, 2.76)	0.103	341 (22)	0.76 (0.56, 1.03)	0.076	0.133
Occupation							
Student or child (not at school)	111 (20)	1		136 (13)	1		
Unemployed or housewife	63 (24)	1.21 (0.70, 2.08)		61 (22)	0.79 (0.34, 1.85)		
Agricultural, fishing or lake related	11 (20)	0.74 (0.29, 1.87)		273 (22)	0.83 (0.39, 1.72)		
Professional or service providers	82 (28)	1.26 (0.77, 2.06)	0.709	103 (25)	0.93 (0.54, 1.62)	0.932	0.473
Maternal tribe							
Central Uganda	127 (25)	1		212 (20)	1		
Other, Ugandan	113 (21)	0.82 (0.52, 1.30)		272 (19)	0.86 (0.59, 1.27)		
Non‐Ugandan, African	26 (25)	**1.77 (1.17, 2.70)**	**0.015**	86 (18)	0.76 (0.44, 1.32)	0.613	0.127
Maternal history of allergies							
No	192 (21)	1		433 (20)	1		
Yes	34 (31)	1.68 (0.89, 3.18)	0.107	71 (15)	0.90 (0.58, 1.41)	0.644	**0.013**
Location of birth							
City	16 (37)	1		12 (21)	1		
Town	34 (28)	0.56 (0.30, 1.02)		57 (24)	0.75 (0.37, 1.52)		
Village	60 (21)	**0.34 (0.18, 0.61)**	**0.004**	397 (21)	0.61 (0.29, 1.28)	0.419	**0.041**
BCG scar							
No	67 (19)	1		228 (19)	1		
Yes	234 (24)	**2.22 (1.24, 3.97)**	**0.010**	345 (19)	1.31 (0.96, 1.79)	0.083	0.601
Lake contact							
Never	72 (18)	1					
Rarely	140 (27)	0.92 (0.50, 1.67)		22 (33)	1		
Once a month	29 (24)	0.78 (0.39, 1.61)					
Once a week	26 (23)	1.04 (0.42, 2.57)	0.896	47 (24)	1.04 (0.64, 1.68)		
Daily/almost daily				385 (22)	0.89 (0.54, 1.48)	0.499	
Bathe in water from lake?							
No	249 (23)	1		25 (36)	1		
Yes	18 (18)	0.75 (0.27, 2.06)	0.558	429 (22)	**0.41 (0.24, 0.71)**	**0.002**	0.172
Hand washing after toilet							
No	19 (12)	1		151 (23)	1		
Yes	248 (25)	4.67 (0.88, 24.8)	0.068	303 (22)	0.78 (0.59, 1.02)	0.068	**0.001**
SWA‐specific IgG4^e^		1.04 (0.86, 1.24)	0.691		**0.77 (0.63, 0.94)**	**0.013**	**0.011**
SEA‐specific IgE^e^		1.32 (0.90, 1.91)	0.135		0.58 (0.29, 1.16)	0.119	0.109
*Sm* infection (KK)							
Uninfected	221 (22)	1		376 (21)	1		
Infected	20 (26)	1.47 (0.76, 2.83)	0.239	127 (16)	**0.68 (0.47, 0.97)**	**0.038**	0.332
*Sm* infection intensity (KK)							
Uninfected	221 (22)	1		376 (21)	1		
Light	15 (38)	**2.39 (1.24, 4.64)**		65 (16)	0.66 (0.43, 1.01)		
Moderate	3 (12)	0.76 (0.22, 2.61)		40 (18)	0.83 (0.52, 1.34)		
Heavy	2 (14)	0.55 (0.05, 6.81)	0.055	22 (12)	0.49 (0.22, 1.14)	0.053	**0.015**
*Sm* infection (PCR)							
Uninfected	188 (21)	1		289 (22)	1		
Infected	48 (25)	**1.57 (1.01, 2.43)**	**0.044**	214 (17)	**0.66 (0.49, 0.89)**	**0.010**	**0.002**
*Sm* infection (CCA)							
Negative	163 (24)	1		114 (27)	1		
Positive	115 (22)	1.19 (0.69, 2.06)	0.517	414 (18)	**0.56 (0.37, 0.83)**	**0.006**	0.184
Malaria treatment, last 12 mo							
No	163 (24)	1		234 (21)	1		
Yes	100 (22)	0.86 (0.52, 1.42)	0.536	323 (18)	1.08 (0.85, 1.38)	0.502	0.730
HIV							
Negative	272 (22)	1		380 (19)	1		
Positive	19 (32)	1.82 (0.56, 5.93)	0.302	98 (25)	1.17 (0.74, 1.85)	0.495	0.440

Associations shown in this table are from adjusted analyses. Full table with crude associations is shown in supplementary Table S1. This table shows only factors that were associated with SPT reactivity (before and/or after adjustment) in either the urban or the rural survey. All other factors that were assessed are listed in the statistical methods section. Significant associations are highlighted in bold. Interaction *P* values are shown to establish whether associations between potential risk factors and SPT reactivity differed, or not, given the setting.

aOR: adjusted odds ratios; CCA: circulating cathodic antigen; KK: Kato‐Katz; PCR: Polymerase Chain Reaction; SEA: Schistosoma egg antigen; SWA: Schistosoma adult worm antigen.

a Number (percentage in parenthesis) of SPT reactive individuals in each category.

b Odds ratios (ORs) and 95% confidence intervals (CI) were adjusted for survey design.

c ORs were adjusted for location of birth, BCG scar, hand washing after toilet use, alcohol use, age and sex.

d ORs were adjusted for HIV infection status, maternal history of allergies, recent malaria treatment, presence/absence of older siblings, age and sex.

e Log10 (concentration+1) transformation applied before analysis.

In addition to the *Sm*‐SPT association, tests for interaction showed that associations between several other risk factors and SPT reactivity differed by survey setting. Being male (*P* = 0.015), maternal history of allergies (*P* = 0.013), SWA‐specific IgG4 (*P* = 0.011) and hand washing (*P* = 0.001) were positively associated with SPT in the urban survey but inversely associated with the same outcome in the rural survey. The inverse association between SPT and being born in a village (compared to town or city) was stronger in the urban compared to rural survey (*P* = 0.041).

Associations with SPT reactivity to individual allergens are summarized in Table S3 **,** and paint a similar picture.

Comparison of models with and without additional adjustment for current *Sm* infection (generally, or categorized by infection intensity) and *Schistosoma*‐specific antibodies did not suggest any mediating role of *Sm* infection in associations between non‐helminth‐related risk factors and SPT reactivity, or in interactions between the rural and urban survey (Table S4A).

### 3.4 Factors associated with allergen‐specific IgE sensitization

Table**** 4 and Table S5 show factors associated with ImmunoCAP IgE sensitization to any of *D pteronyssinus*,* A hypogaea* or *B germanica* extracts. In the urban survey, the presence of younger siblings and SWA‐specific IgG were associated with asIgE sensitization. Rural participants who washed hands after toilet use, slept under a mosquito net and/or had recently been treated for malaria were less likely to be asIgE sensitized. Engaging in agricultural/fishing/lake‐related activities or being unemployed, *Sm* infection (KK) and intensity, and elevated SWA‐specific IgE increased the odds of asIgE sensitization.

**Table 4:** Factors associated with IgE sensitization (ImmunoCAP IgE > 0.35 kU/L) to any of *D pteronyssinus*,* A hypogaea* or *B germanica*

Factor	Urban			Rural			Interaction *P*
	N (%)^a^	aOR (95% CI)^b^, ^d^	*P*	N (%)^a^	aOR (95% CI)^b^, ^c^	*P*	
Age		0.99 (0.98, 1.01)	0.547		1.01 (0.98, 1.03)	0.589	0.728
Sex							
Male	47 (48)	1		241 (64)	1		
Female	101 (41)	0.77 (0.51, 1.15)	0.200	196 (49)	0.69 (0.42, 1.14)	0.140	0.407
Younger siblings (Yes/No)							
No	27 (33)	1		61 (62)	1		
Yes	106 (46)	**2.07 (1.07, 4.01)**	**0.030**	313 (56)	0.76 (0.53, 1.09)	0.129	**0.008**
Occupation							
Student or child (not at school)	68 (48)	1		64 (51)	1		
Unemployed or housewife	34 (40)	0.70 (0.31, 1.60)		58 (54)	**2.05 (1.38, 3.03)**		
Agricultural, fishing or lake related	4 (36)	0.56 (0.13, 2.46)		251 (61)	**1.87 (1.04, 3.37)**		
Professional or service providers	27 (38)	0.61 (0.26, 1.43)	0.725	61 (47)	1.38 (0.72, 2.66)	**0.014**	0.148
Lake contact							
Never	39 (45)	1					
Rarely	69 (42)	0.98 (0.52, 1.85)		8 (42)	1		
Once a month	11 (35)	0.74 (0.38, 1.41)					
Once a week	14 (50)	1.32 (0.49, 3.55)	0.835	25 (44)	0.82 (0.23, 2.89)		
Daily/almost daily				343 (59)	1.64 (0.45, 5.90)	0.174	
Bathe in water from lake?							
No	121 (42)	1		15 (63)	1		
Yes	12 (57)	1.86 (0.50, 6.87)	0.331	361 (57)	0.42 (0.15, 1.11)	0.078	0.065
Hand washing after toilet							
No	15 (39)	1		148 (71)	1		
Yes	117 (43)	1.36 (0.69, 2.66)	0.344	228 (50)	**0.43 (0.30, 0.61)**	**<0.001**	**0.003**
SWA‐specific IgE^e^		2.95 (0.51, 17.2)	0.214		**6.17 (2.79, 13.6)**	**<0.001**	0.459
SWA‐specific IgG4^e^		1.01 (0.85, 1.19)	0.932		1.07 (0.89, 1.27)	0.470	0.433
SEA‐specific IgG4^e^		1.11 (0.97, 1.25)	0.102		1.07 (0.95, 1.21)	0.227	0.704
SWA‐specific IgG^e^		**3.33 (1.12, 9.86)**	**0.031**		1.53 (0.82, 2.86)	0.177	0.340
SEA‐specific IgG^e^		1.77 (0.63, 4.96)	0.260		1.43 (0.88, 2.30)	0.138	0.796
*S mansoni* infection (KK)							
Uninfected	119 (44)	1		271 (55)	1		
Infected	6 (43)	1.06 (0.34, 3.35)	0.910	118 (63)	**1.52 (1.19, 1.94)**	**0.002**	0.180
*S mansoni* infection intensity (KK)							
Uninfected	119 (44)	1		271 (55)	1		
Light	2 (29)	0.66 (0.11, 4.04)		54 (57)	**1.74 (1.18, 2.54)**		
Moderate	2 (50)	1.20 (0.16, 8.79)		35 (66)	0.94 (0.46, 1.90)		
Heavy	2 (67)	2.32 (0.13, 40.9)	0.662	29 (73)	2.37 (0.71, 7.83)	**0.028**	0.536
Any nematode infection^f^							
No	109 (42)	1		281 (54)	1		
Yes	16 (62)	2.34 (0.76, 7.19)	0.130	108 (66)	1.53 (0.94, 2.49)	0.084	0.287
Slept under mosquito net last night?							
No	35 (45)	1		203 (62)	1		
Yes	97 (42)	0.93 (0.52, 1.66)	0.958	172 (52)	**0.63 (0.41, 0.97)**	**0.037**	0.316
Malaria treatment, last 12 mo							
No	83 (45)	1		202 (63)	1		
Yes	48 (39)	0.78 (0.46, 1.35)	0.365	221 (51)	**0.52 (0.34, 0.81)**	**0.005**	0.185

Associations shown in this table are from adjusted analyses. Full table with crude associations is shown in supplementary Table S1. This table shows only factors that were associated with IgE sensitization (before and/or after adjustment) in either the urban or the rural survey. All other factors that were assessed are listed in the statistical methods section. Significant associations are highlighted in bold. Interaction *P* values are shown to denote whether tests for interaction showed statistical evidence for urban‐rural differences in associations with IgE sensitization, or not.

aOR: adjusted odds ratios; KK: Kato‐Katz; SWA: Schistosoma adult worm antigen; SEA: Schistosoma egg antigen.

a Number (percentage in parenthesis) of IgE sensitized individuals in each category.

b Odds ratios (ORs) and 95% confidence intervals (CI) adjusted for survey design.

c All ORs were adjusted for hand washing after toilet use, mosquito net use, malaria treatment, age and sex.

d All ORs were adjusted for age and sex.

e Log10 (concentration+1) transformation applied before analysis.

f Infection with any of *Ascaris lumbricoides*,* Trichuris trichiura (assessed by KK), Necator americanus*,* Strongyloides stercoralis* (assessed by PCR).

The presence of younger siblings (interaction *P* = 0.008) and hand washing (interaction *P* = 0.003) were associated with reduced odds of asIgE sensitization in the rural but not the urban survey (Table**** 4). Adjusting for *Sm* infection in multivariable analysis models did not suggest a mediating role for *Sm* in these rural‐urban differences (Table S4B).

Table S6 summarizes factors associated with ImmunoCAP asIgE sensitization to individual allergens: *Schistosoma*‐specific antibody levels and helminth infections were the predominant risk factors in both surveys. Hygiene practices (washing and bathing) reduced the odds of sensitization in the rural but not urban survey.

### 3.5 Factors associated with clinical allergy‐related outcomes

Factors associated with self‐reported recent wheeze, urticarial rash and rhinitis are shown in Table S7. Risk factors for visible flexural dermatitis could not be assessed because it was rare in both settings. In the urban survey, the presence of older siblings, handwashing before eating, SWA‐specific IgG and SEA‐specific IgG were inversely associated with wheezing. In the rural survey, female sex and presence of any nematode infection were inversely associated with wheezing, while increasing age, SWA‐specific IgG, SEA‐specific IgG and paternal history of allergies increased the odds of wheezing. Non‐Ugandan paternal tribe (interaction *P* < 0.001) increased the odds of wheezing in the urban but not rural survey, while SWA‐specific IgG (*P* < 0.001) and SEA‐specific IgG (*P* = 0.001) were positively associated with wheezing in the rural but not the urban survey.

Urban individuals who received any anthelminthic treatment in the previous 12 months were more likely to report urticarial rash. In the rural survey, increasing age, maternal history of allergies, SEA‐specific IgE and recent malaria treatment were associated with urticaria. The association between SEA‐specific IgE and urticaria was positive in the rural but not urban survey (interaction *P* = 0.022). No other significant interactions were observed.

Maternal and paternal history of allergies, and HIV infection were associated with rhinitis in the urban survey. The following were risk factors for rhinitis in the rural survey: increasing age, presence of older siblings, being born in a city (compared to town or village) and bathing in lakewater. The positive association between HIV and rhinitis was stronger in the urban compared to the rural survey (interaction *P* = 0.028). No other significant interactions were observed.

We did not find any evidence to suggest that current *Sm* infection influenced associations between non‐helminth‐related risk factors and clinical allergy‐related outcomes, and interactions between the rural and urban survey (Table S4, C‐E).

## 4 DISCUSSION

We show risk factors for allergy‐related outcomes in proximate Ugandan rural and urban settings. The rural setting was characterized by a significantly higher prevalence of *Sm* and nematode infections compared to the urban setting. The prevalence of SPT reactivity and visible flexural dermatitis was lower, and that of asIgE sensitization and urticaria higher, in the rural compared to urban setting. Risk factors for these outcomes differed by setting. We investigated the hypothesis that rural‐urban differences in risk factors for allergy were attributable to differences in current *Sm* exposure. Despite observations that the rural environment (and higher intensity *Sm* infection within it) was associated with reduced odds of SPT reactivity, statistical analyses did not confirm a mediating role for current *Sm* infection in the rural‐urban differences, implying that other exposures may play important roles. Similarly, rural‐urban differences in associations with clinical allergy outcomes could not categorically be attributed to differences in current *Sm* infection between the two settings.

Our rural and urban settings were atypical. Observations in the rural survey are against a backdrop of three years of well‐organized community‐level anthelminthic intervention^25^ that led to a decline in helminth intensity in both standard and intensive treatment arms, but had no effect on overall *Sm* prevalence.^26^ Before analysis of risk factors, we confirmed a lack of effect of the intensive (compared with standard) anthelminthic treatment on allergy‐related outcomes. The urban survey was done in the unusual context of a setting with considerable exposure to light *Sm* infection (inferred from 44% urine CCA positivity). However, this enabled us to adjust for *Sm* infection in both settings and hence explore the role of *Sm* in interactions between the settings. Recruitment of participants in the urban survey was done after conclusion of the rural survey; however, this is unlikely to account for observed urban‐rural differences in allergy risk factors, as both surveys were conducted by the same research team, and covered approximately 1 year (so any seasonal effects were approximately matched). Another potential limitation was the large number of statistical tests, increasing likelihood of chance findings. However, we were cautious to look for patterns of association rather than interpreting individual results equally.

In keeping with the “old friends” hypothesis^1^ and observations from several studies,^37^, ^38^, ^39^ SPT reactivity was less prevalent in the helminth‐endemic rural setting and was inversely associated with helminth infections in the same setting. The only exception was *Trichuris trichiura* infection, which was weakly positively associated with *Dermatophagoides* SPT (Table S3). This lone observation was also manifest in the same communities in a baseline household survey 3 years earlier,^24^ although no other helminth species were associated with SPT then. The current observations beg further investigation into the impact of anthelminthic treatment on SPT‐helminth associations in a helminth‐endemic setting. In mice, allergic airway inflammation is increased during acute *Sm* infection but reduces drastically with progression to chronic infection.^40^ In our urban setting, light *Sm* infection was positively associated with SPT reactivity while moderate and heavy infections were inversely associated with the same outcome (Table**** 3). “Helminth‐related” behavioural characteristics were also inversely associated with SPT reactivity in the rural survey. It is plausible that in these fishing communities, frequent lake contact, bathing in lakewater and handwashing, for example, increase the risk for *Sm* infection through contact with infected snails. Indeed, these characteristics were strongly associated with *Sm* infection (*P* < 0.001). However, the same characteristics were also inversely associated with asIgE sensitization in the rural survey but not in the urban survey.

As discussed earlier, Pinot de Moira and colleagues’ study in a Ugandan village found that hookworm infection abrogated the predicted association between *Dermatophagoides*‐specific IgE and basophil histamine release.^22^ We postulated that the rural setting might interfere with the link between atopic sensitization (asIgE, SPT) and clinical outcomes (reported wheeze and rhinitis) through high helminth exposure. Indeed, we found that associations between asIgE or SPT sensitization and clinical outcomes were weak among participants from the rural compared to the urban setting. However, statistical analyses did not suggest that this difference was mediated by current *Sm* infection. Furthermore, the PAF for SPT associated with asIgE in both the helminth‐endemic rural survey and in the urban survey was high, and adjusting for *Sm* or *Schistosoma*‐specific antibodies had no effect on this association.

Allergen‐specific IgE sensitization, particularly to cockroach, was more prevalent in the rural compared to the urban setting, possibly due to the higher helminth prevalence in the former. Additionally, helminth infections and *Schistosoma*‐specific antibody levels were positively associated with asIgE in both surveys. Our immunoassays measured IgE sensitization to crude allergen extracts; these may contain cross‐reactive components that are conserved in several helminth antigens,^41^, ^42^, ^43^, ^44^, ^45^, ^46^ explaining the above associations. These cross‐reactive components may be less effective at mediating the effector phase of the allergic response, explaining the lower prevalence of SPT reactivity in the helminth‐endemic rural survey.

Associations with wheeze and rhinitis should be interpreted with caution, because these outcomes were relatively rare. Furthermore, reported wheeze can easily be misclassified in these populations, because there is no direct translation of the word “wheeze” in the local languages.^24^, ^47^ Nonetheless, rural‐urban differences in the risk factors for these outcomes were visible. For example, while *Schistosoma*‐specific antibody levels were inversely associated with wheezing in the urban survey, the reverse was true in the rural setting. Urticarial rash was a more common outcome, particularly in the helminth‐endemic rural survey, where it may be indicative of parasite‐induced skin allergy^48^ and reaction to parasite antigens following anthelminthic treatment.^49^ Support for these deductions comes from our observations that recent anthelminthic treatment (urban survey) and SEA‐specific IgE (rural survey) were associated with urticaria.

In conclusion, we show that risk factors for allergy‐related outcomes differ between rural and urban communities in this tropical setting. However, our analyses did not confirm a role for current helminth (*Sm*) infection as the primary mechanism of the observed effect modification between the two settings, despite indicative trends. Differences in other environmental exposures may contribute significantly.

## CONFLICT OF INTEREST

The authors declare no conflict of interest.

## AUTHOR CONTRIBUTIONS

AME conceived the LaVIISWA study and the urban survey. GN, AME, MY and RvR designed the laboratory studies. GN, JK, JN and SV performed the laboratory experiments. AME, RES, MN, PNK, JT, CZ, RK, EN, HM and CN led and participated in field and clinic procedures. GN analysed the results with significant input from LL, ELW, HM, MY and AME. GN wrote the manuscript, with all authors contributing to the interpretation of the results, and revision and approval of the final manuscript. GN is the guarantor of the article.
