# Supplementary material for: Do helminth infections underpin urban‐rural differences in risk factors for allergy‐related outcomes?
Source: Clin Exp Allergy. 2019 Jan 25;49(5):663–76. doi: 10.1111/cea.13335 (PMC6518997; doi:10.1111/cea.13335)
Supplement: Supplementary file 1 [file CEA-49-663-s001.docx]

**Do helminth infections underpin urban-rural differences in risk factors for allergy-related outcomes?**

**SUPPLEMENTARY INFORMATION**

1. **Supplementary methods**
   1. *S. mansoni adult worm (SWA)- and egg (SEA)-specific IgE and IgG4 ELISA*

All but the first 2 columns of 4HBX Immulon (Thermo Scientific, NY, USA) 96-well plates were coated with 50μl of SWA [8 μg/ml] or SEA [2.4 μg/ml] (purchased from Professor Michael J Doenhoff, University of Nottingham) in bicarbonate (Na_2_CO_3_ + NaHCO_3_) buffer (0.1M, pH 9.6). Two-fold dilutions of human IgE (Calbiochem, Beeston, UK) or IgG4 (Sigma-Aldrich) standard, diluted in bicarbonate buffer, were added to the first 2 columns of each plate to form standard curves. The plates were then incubated overnight at 4^0^C. Plates were washed with phosphate-buffered saline (PBS 1X)-tween 20 solution, blocked with 150μl of 1% skimmed milk diluted in PBS-Tween 20 at room temperature (RT), and incubated overnight at 4^0^C with 50μl of plasma samples diluted 1/20 (IgE assay) or 1/200 (IgG4 assay) with 0.1% skimmed milk in PBS-Tween 20 (assay buffer). Plates were washed and antibody binding detected by incubating the plates overnight at 4^0^C with 0.5μg/ml of biotinylated monoclonal mouse anti-human IgE or IgG4 (BD Pharmingen™). This was followed by a 1 hour incubation with a streptavidin-Horseradish Peroxidase (strep-HRP) conjugate (Mast Group Ltd, Bootle, UK), diluted 1/4000 with assay buffer, at RT. Plates were developed by addition of 100μl of o-phenylenediamine (Sigma-Aldrich) and reactions stopped after 30 minutes with 25μl of 2M Sulphuric acid. Optical density values were measured at 490nm (reference wavelength 630nm) on a 96-well plate ELISA reader. IgE or IgG4 concentrations (ng/ml) were interpolated from standard curves using a five-parameter curve fit using Gen5 data collection and analysis software (BioTek Instruments Inc, Vermont, Winooski, USA).

- 1. *S. mansoni adult worm (SWA)- and egg (SEA)-specific IgG ELISA*

All but the first 2 columns of 4HX Immulon (VWR, UK, Cat No 735-0465) 96-well plates were coated with 50μl of SWA [8 μg/ml] or SEA [2.4 μg/ml] (purchased from Professor Michael J Doenhoff, University of Nottingham) in bicarbonate (Na_2_CO_3_ + NaHCO_3_) buffer (0.1M, pH 9.6). Two-fold dilutions of human IgG (Sigma-Aldrich), diluted in bicarbonate buffer, were added to the first 2 columns of each plate to form standard curves. The plates were then incubated overnight at 4^0^C. Plates were washed with phosphate-buffered saline (PBS)-tween 20 solution, blocked with 150μl of 1% skimmed milk diluted in PBS-Tween 20 at room temperature (RT), and incubated overnight at 4^0^C with 50μl of plasma samples diluted 1/3000 with 0.1% skimmed milk in PBS-Tween 20 (assay buffer). Plates were washed and antibody binding detected by incubating the plates for 1 hour at RT with 0.5μg/ml of polyclonal rabbit anti- human IgG/HRP (Dako, Denmark). Plates were developed by addition of 100μl of o-phenylenediamine (Sigma-Aldrich) and reactions stopped after 30 minutes with 25μl of 2M Sulphuric acid. Optical density values were measured at 490nm (reference wavelength 630nm) on a 96-well plate ELISA reader. IgG concentrations (ng/ml) were interpolated from standard curves using a five-parameter curve fit using Gen5 data collection and analysis software (BioTek Instruments Inc, Vermont, Winooski, USA).

1. **Supplementary tables**

**Table S1. Crude versus *Sm*-adjusted associations between allergy-related outcomes**

|  |  | | **SPT** | | **WHEEZE** | | **RHINITIS** | | **URTICARIA** | |
| --- | --- | --- | --- | --- | --- | --- | --- | --- | --- | --- |
|  |  | | Unadjusted | Adjusted for *Sm* (CCA)* | Unadjusted | Adjusted for *Sm* (CCA)* | Unadjusted | Adjusted for *Sm* (CCA)* | Unadjusted | Adjusted for *Sm* (CCA)* |
| **asIgE** | **URBAN** | OR (95% CI) | **21.4 (10.2, 44.6)** | **23.6 (10.2, 54.4)** | 5.5 (0.4, 68.6) | 4.4 (0.4, 47.3) | **3.7 (1.2, 11.9)** | **3.7 (1.1, 12.1)** | 3.7 (0.8, 16.2) | 3.4 (0.8, 14.5) |
|  |  | p value | **<0.001** | **<0.001** | 0.171 | 0.210 | **0.028** | **0.035** | 0.075 | 0.092 |
|  | **RURAL** | OR (95% CI) | **10.3 (5.3, 19.8)** | **10.1 (4.9, 20.9)** | **3.9 (1.3, 11.5)** | 3.0 (0.9, 9.5) | 1.1 (0.5, 2.6) | 0.99 (0.3, 2.8) | 0.9 (0.6, 1.3) | 0.9 (0.6, 1.3) |
|  |  | p value | **<0.001** | **<0.001** | **0.015** | 0.061 | 0.793 | 0.979 | 0.651 | 0.659 |
|  |  | Interaction p | 0.127 | 0.074 | 0.792 | 0.817 | 0.081 | 0.073 | 0.056 | 0.089 |
| **SPT** | **URBAN** | OR (95% CI) |  |  | 2.2 (0.6, 8.1) | 1.7 (0.5, 6.1) | **6.5 (3.4, 12.5)** | **5.9 (3.1, 11.4)** | **2.2 (1.6, 2.8)** | **2.3 (1.7, 3.0)** |
|  |  | p value |  |  | 0.211 | 0.366 | **<0.001** | **<0.001** | **<0.001** | **<0.001** |
|  | **RURAL** | OR (95% CI) |  |  | **3.0 (1.8, 5.1)** | **3.6 (1.9, 6.8)** | **2.6 (1.7, 3.9)** | **2.7 (1.7, 4.1)** | 1.2 (0.9, 1.6) | 1.03 (0.7, 1.4) |
|  |  | p value |  |  | **<0.001** | **<0.001** | **<0.001** | **<0.001** | 0.243 | 0.845 |
|  |  | Interaction p |  |  | 0.647 | 0.281 | **0.019** | **0.037** | **0.005** | **0.001** |
| **WHEEZE** | **URBAN** | OR (95% CI) |  |  |  |  | **7.4 (1.7, 33.2)** | **7.1 (1.5, 32.8)** | **4.9 (1.1, 21.7)** | **6.1 (1.4, 27.3)** |
|  |  | p value |  |  |  |  | **0.011** | **0.015** | **0.035** | **0.020** |
|  | **RURAL** | OR (95% CI) |  |  |  |  | **11.9 (5.7, 24.9)** | **14.9 (6.8, 32.5)** | 1.4 (0.6, 3.3) | 1.2 (0.6, 2.7) |
|  |  | p value |  |  |  |  | **<0.001** | **<0.001** | 0.403 | 0.588 |
|  |  | Interaction p |  |  |  |  | 0.557 | 0.375 | 0.127 | 0.056 |
| **RHINITIS** | **URBAN** | OR (95% CI) |  |  |  |  |  |  | **9.6 (5.6, 16.4)** | **10.4 (5.4, 19.7)** |
|  |  | p value |  |  |  |  |  |  | **<0.001** | **<0.001** |
|  | **RURAL** | OR (95% CI) |  |  |  |  |  |  | 0.7 (0.3, 1.6) | 0.6 (0.2, 1.7) |
|  |  | p value |  |  |  |  |  |  | 0.429 | 0.321 |
|  |  | Interaction p |  |  |  |  |  |  | **<0.001** | **<0.001** |
| *Odds ratios (ORs) and p values were obtained from survey design-adjusted analyses. Visible flexural dermatitis was not assessed because it was rare. Significant associations are highlighted in bold.*  ** Adjusting for other Sm infection variables (KK / PCR), Sm infection intensity (KK) and SWA- and SEA-specific antibodies paints a similar picture.*  ***asIgE:*** *ImmunoCAP IgE sensitisation to any of D. pteronyssinus, A. hypogaea, or B. germanica on ImmunoCAP;* ***SPT****: skin prick test reactivity to any of Dermatophagoides mix, B. tropicalis or B. germanica;* ***CCA:*** *Circulating Cathodic Antigen* | | | | | | | | | | |

**Table S2. Crude and adjusted associations with SPT reactivity to any of *Dermatophagoides* mix, *B. tropicalis* or *B. germanica***

|  |  | UNADJUSTED ANALYSIS | | | | | |  | ADJUSTED ANALYSIS | | | | |
| --- | --- | --- | --- | --- | --- | --- | --- | --- | --- | --- | --- | --- | --- |
|  |  |  | | | | | |  |  | | | | |
|  |  | **URBAN** | | | **RURAL** | | |  | **URBAN** |  | **RURAL** |  |  |
| Factor |  | N (%)^≠^ | OR (95% CI)¶ | p | N (%)^≠^ | OR (95% CI)¶ | p |  | OR (95% CI) ¶# | p | OR (95% CI)¶§ | p | Interaction p |
|  |  |  |  |  |  |  |  |  |  |  |  |  |  |
| Age |  |  | **1.01 (1.00, 1.02)** | **0.005** |  | **1.02 (1.01, 1.03)** | **<0.001** |  | **1.02 (1.00, 1.03)** | **0.035** | **1.02 (1.00, 1.03)** | **0.015** | 0.384 |
|  |  |  |  |  |  |  |  |  |  |  |  |  |  |
| Sex |  |  |  |  |  |  |  |  |  |  |  |  |  |
| Male |  | 132 (26) | 1 |  | 285 (18) | 1 |  |  | 1 |  | 1 |  |  |
| Female |  | 170 (21) | 0.77 (0.59, 1.02) | 0.068 | 291 (20) | 1.00 (0.74, 1.37) | 0.979 |  | 0.71 (0.49, 1.02) | 0.061 | 1.09 (0.79, 1.52) | 0.558 | **0.015** |
| Older siblings (Yes/No) |  |  |  |  |  |  |  |  |  |  |  |  |  |
| No |  | 73 (22) | 1 |  | 113 (24) | 1 |  |  | 1 |  | 1 |  |  |
| Yes |  | 194 (23) | 1.05 (0.78, 1.42) | 0.691 | 341 (22) | **0.75 (0.58, 0.98)** | **0.036** |  | 1.58 (0.90, 2.76) | 0.103 | 0.76 (0.56, 1.03) | 0.076 | 0.133 |
| Occupation |  |  |  |  |  |  |  |  |  |  |  |  |  |
| Student or child (not at school) |  | 111 (20) | 1 |  | 136 (13) | 1 |  |  | 1 |  | 1 |  |  |
| Unemployed or housewife |  | 63 (24) | 1.22 (0.92, 1.63) |  | 61 (22) | **1.68 (1.09, 2.58)** |  |  | 1.21 (0.70, 2.08) |  | 0.79 (0.34, 1.85) |  |  |
| Agricultural, fishing or lake related |  | 11 (20) | 0.98 (0.44, 2.21) |  | 273 (22) | **1.78 (1.41, 2.25)** |  |  | 0.74 (0.29, 1.87) |  | 0.83 (0.39, 1.72) |  |  |
| Professional or service providers |  | 82 (28) | 1.57 (1.16, 2.14) | 0.059 | 103 (25) | **1.96 (1.25, 3.06)** | **<0.001** |  | 1.26 (0.77, 2.06) | 0.709 | 0.93 (0.54, 1.62) | 0.932 | 0.473 |
| Maternal tribe |  |  |  |  |  |  |  |  |  |  |  |  |  |
| Central Uganda |  | 127 (25) | 1 |  | 212 (20) | 1 |  |  | 1 |  | 1 |  |  |
| Other, Ugandan |  | 113 (21) | 0.78 (0.59, 1.03) |  | 272 (19) | 1.03 (0.76, 1.35) |  |  | 0.82 (0.52, 1.30) |  | 0.86 (0.59, 1.27) |  |  |
| Non-Ugandan, African |  | 26 (25) | 1.01 (0.59, 1.71) | 0.179 | 86 (18) | 1.03 (0.79, 1.32) | 0.977 |  | **1.77 (1.17, 2.70)** | **0.015** | 0.76 (0.44, 1.32) | 0.613 | 0.127 |
| Maternal history of allergies |  |  |  |  |  |  |  |  |  |  |  |  |  |
| No |  | 192 (21) | 1 |  | 433 (20) | 1 |  |  | 1 |  | 1 |  |  |
| Yes |  | 34 (31) | 1.65 (0.95, 2.86) | 0.073 | 71 (15) | **0.75 (0.58, 0.95)** | **0.018** |  | 1.68 (0.89, 3.18) | 0.107 | 0.90 (0.58, 1.41) | 0.644 | **0.013** |
| Location of birth |  |  |  |  |  |  |  |  |  |  |  |  |  |
| City |  | 16 (37) | 1 |  | 12 (21) | 1 |  |  | 1 |  | 1 |  |  |
| Town |  | 34 (28) | 0.64 (0.38, 1.09) |  | 57 (24) | 0.90 (0.42, 1.95) |  |  | 0.56 (0.30, 1.02) |  | 0.75 (0.37, 1.52) |  |  |
| Village |  | 60 (21) | **0.44 (0.28, 0.68**) | **0.005** | 397 (21) | 0.89 (0.49, 1.61) | 0.932 |  | **0.34 (0.18, 0.61)** | **0.004** | 0.61 (0.29, 1.28) | 0.419 | **0.041** |
| BCG scar |  |  |  |  |  |  |  |  |  |  |  |  |  |
| No |  | 67 (19) | 1 |  | 228 (19) | 1 |  |  | 1 |  | 1 |  |  |
| Yes |  | 234 (24) | **1.39 (1.05, 1.83)** | **0.021** | 345 (19) | 1.07 (0.89, 1.28) | 0.444 |  | **2.22 (1.24, 3.97)** | **0.010** | 1.31 (0.96, 1.79) | 0.083 | 0.601 |
| Lake contact |  |  |  |  |  |  |  |  |  |  |  |  |  |
| Never |  | 72 (18) | 1 |  |  |  |  |  | 1 |  |  |  |  |
| Rarely |  | 140 (27) | **1.64 (1.36, 1.99)** |  | 22 (33) | 1 |  |  | 0.92 (0.50, 1.67) |  | 1 |  |  |
| Once a month |  | 29 (24) | 1.42 (0.99, 2.02) |  |  |  |  |  | 0.78 (0.39, 1.61) |  |  |  |  |
| Once a week |  | 26 (23) | 1.37 (0.88, 2.13) | **0.021** | 47 (24) | 0.73 (0.44, 1.22) |  |  | 1.04 (0.42, 2.57) | 0.896 | 1.04 (0.64, 1.68) |  |  |
| Daily/ almost daily |  |  |  |  | 385 (22) | **0.65 (0.44, 0.96)** | **0.033** |  |  |  | 0.89 (0.54, 1.48) | 0.499 |  |
| Bathe in water from lake? |  |  |  |  |  |  |  |  |  |  |  |  |  |
| No |  | 249 (23) | 1 |  | 25 (36) | 1 |  |  | 1 |  | 1 |  |  |
| Yes |  | 18 (18) | 0.74 (0.32, 1.67) | 0.447 | 429 (22) | **0.38 (0.18, 0.80)** | **0.013** |  | 0.75 (0.27, 2.06) | 0.558 | **0.41 (0.24, 0.71)** | **0.002** | 0.172 |
| Hand washing after toilet |  |  |  |  |  |  |  |  |  |  |  |  |  |
| No |  | 19 (12) | 1 |  | 151 (23) | 1 |  |  | 1 |  | 1 |  |  |
| Yes |  | 248 (25) | **2.49 (1.77, 3.49)** | **<0.001** | 303 (22) | 0.88 (0.68, 1.13) | 0.309 |  | 4.67 (0.88, 24.8) | 0.068 | 0.78 (0.59, 1.02) | 0.068 | **0.001** |
|  |  |  |  |  |  |  |  |  |  |  |  |  |  |
| SWA-specific IgG4* |  |  | 1.03 (0.94, 1.13) | 0.449 |  | 0.87 (0.75, 1.02) | 0.093 |  | 1.04 (0.86, 1.24) | 0.691 | **0.77 (0.63, 0.94)** | **0.013** | **0.011** |
| SEA-specific IgE* |  |  | **1.47 (1.02, 2.11)** | **0.038** |  | 1.03 (0.58, 1.81) | 0.923 |  | 1.32 (0.90, 1.91) | 0.135 | 0.58 (0.29, 1.16) | 0.119 | 0.109 |
|  |  |  |  |  |  |  |  |  |  |  |  |  |  |
| *Sm* infection (KK) |  |  |  |  |  |  |  |  |  |  |  |  |  |
| Uninfected |  | 221 (22) | 1 |  | 376 (21) | 1 |  |  | 1 |  | 1 |  |  |
| Infected |  | 20 (26) | 1.22 (0.62, 2.41) | 0.552 | 127 (16) | **0.69 (0.53, 0.91)** | **0.010** |  | 1.47 (0.76, 2.83) | 0.239 | **0.68 (0.47, 0.97)** | **0.038** | 0.332 |
| *Sm* infection intensity (KK) |  |  |  |  |  |  |  |  |  |  |  |  |  |
| Uninfected |  | 221 (22) | 1 |  | 376 (21) | 1 |  |  | 1 |  | 1 |  |  |
| Light |  | 15 (38) | **2.2 (1.09, 4.46)** |  | 65 (16) | **0.71 (0.53, 0.95)** |  |  | **2.39 (1.24, 4.64)** |  | 0.66 (0.43, 1.01) |  |  |
| Moderate |  | 3 (12) | 0.48 (0.07, 3.03) |  | 40 (18) | 0.76 (0.49, 1.18) |  |  | 0.76 (0.22, 2.61) |  | 0.83 (0.52, 1.34) |  |  |
| Heavy |  | 2 (14) | 0.58 (0.13, 2.75) | 0.772 | 22 (12) | **0.55 (0.34, 0.91)** | **0.017** |  | 0.55 (0.05, 6.81) | 0.055 | 0.49 (0.22, 1.14) | 0.053 | **0.015** |
| *Sm* infection (PCR) |  |  |  |  |  |  |  |  |  |  |  |  |  |
| Uninfected |  | 188 (21) | 1 |  | 289 (22) | 1 |  |  | 1 |  | 1 |  |  |
| Infected |  | 48 (25) | 1.26 (0.94, 1.70) | 0.117 | 214 (17) | **0.73 (0.55, 0.96)** | **0.031** |  | **1.57 (1.01, 2.43)** | **0.044** | **0.66 (0.49, 0.89)** | **0.010** | **0.002** |
| *Sm* infection (CCA) |  |  |  |  |  |  |  |  |  |  |  |  |  |
| Negative |  | 163 (24) | 1 |  | 114 (27) | 1 |  |  | 1 |  | 1 |  |  |
| Positive |  | 115 (22) | 0.89 (0.64, 1.26) | 0.508 | 414 (18) | **0.59 (0.44, 0.80)** | **0.001** |  | 1.19 (0.69, 2.06) | 0.517 | **0.56 (0.37, 0.83)** | **0.006** | 0.184 |
| Malaria treatment, last 12months |  |  |  |  |  |  |  |  |  |  |  |  |  |
| No |  | 163 (24) | 1 |  | 234 (21) | 1 |  |  | 1 |  | 1 |  |  |
| Yes |  | 100 (22) | 0.91 (0.72, 1.16) | 0.461 | 323 (18) | **0.82 (0.68, 0.97)** | **0.027** |  | 0.86 (0.52, 1.42) | 0.536 | 1.08 (0.85, 1.38) | 0.502 | 0.730 |
| HIV |  |  |  |  |  |  |  |  |  |  |  |  |  |
| Negative |  | 272 (22) | 1 |  | 380 (19) | 1 |  |  | 1 |  | 1 |  |  |
| Positive |  | 19 (32) | 1.61 (0.97, 2.64) | 0.060 | 98 (25) | **1.47 (1.04, 2.08)** | **0.031** |  | 1.82 (0.56, 5.93) | 0.302 | 1.17 (0.74, 1.85) | 0.495 | 0.440 |
|  | | | | | | | | | | | | | |
| *Table shows only factors that were associated with SPT reactivity (before and/or after adjustment) in either the urban or the rural survey. All other factors that were assessed are listed in the statistical methods section. Significant associations are highlighted in bold. Interaction p values are shown to denote whether tests for interaction, using the adjusted model, showed statistical evidence for urban-rural differences in associations with SPT reactivity, or not.*  *≠Number (percentage in parenthesis) of SPT reactive individuals in each category*  *¶Odds ratios (ORs) and 95% confidence intervals (CI) were adjusted for survey design*  *#ORs were adjusted for location of birth, BCG scar, hand washing after toilet use, alcohol use, age and sex.*  *§ORs were adjusted for HIV infection status, maternal history of allergies, recent malaria treatment, presence/absence of older siblings, age and sex.*  **Log_10_ (concentration+1) transformation applied before analysis.*  *KK: Kato-Katz; PCR: Polymerase Chain Reaction; CCA: Circulating Cathodic Antigen; SWA: Schistosoma adult worm antigen; SEA: Schistosoma egg antigen.* | | | | | | | | | | | | | |

**Table S3. Summary of risk factors for SPT reactivity to individual allergen extracts**

|  |  | **URBAN SURVEY** | |  | **RURAL SURVEY** | |
| --- | --- | --- | --- | --- | --- | --- |
|  |  |  |  |  |  |  |
| **Outcome** |  | **Factors positively associated with outcome** | **Factors inversely associated with outcome** |  | **Factors positively associated with outcome** | **Factors inversely associated with outcome** |
|  |  |  |  |  |  |  |
| SPT reactivity, *Dermatophagoides* mix |  | - *S. mansoni* infection [PCR] (p=0.062) and intensity (p for trend=0.007) - Presence of older siblings (p=0.005) | - Female gender (p=0.013) - Recent malaria treatment (p<0.001) |  | - *T. trichiura* infection (p=0.046) - Age (p=0.015) - Type of footwear (sandals or shoes, versus bare feet) [p=0.034] | - *S. mansoni* infection [PCR] (p<0.001) - *S. mansoni* infection [KK] (p=0.001) - *S. mansoni* infection intensity [KK] (p<0.001) - SWA-specific IgG4 (p=0.001) - Bathing in lake water (p=0.016) - SEA-specific IgG (p=0.046) |
|  |  |  |  |  |  |  |
| SPT reactivity, *B. tropicalis* |  | - Age (p=0.032) - Maternal history of allergies (p=0.004) - Hand washing after toilet use (p=0.013) - SEA-specific IgE (p=0.012) | - Paternal tribe (p=0.001) |  | - | - *S. mansoni* infection [KK] (p=0.016) - *S. mansoni* infection intensity [KK] (p=0.006) - SEA-specific IgG (p=0.012) - SWA-specific IgG (p=0.041) - SEA-specific IgG4 (p=0.009) - Bathing in lake water (p<0.001) - Presence of older siblings (p=0.034) - Daily contact with lake (versus weekly or rarely) [p for trend =0.026] |
|  |  |  |  |  |  |  |
| SPT reactivity, *B. germanica* |  | - BCG scar (p=0.017) - Maternal history of allergies (p=0.019) - Presence of older siblings (p=0.033) - SWA-specific IgG4 (p=0.046) | Location of birth (p for trend=0.049) |  | - Age (p<0.001) | - Presence of older siblings (p=0.043) |
|  | | | | | | |
| *P values are from adjusted analyses, conducted as described in the main text. The following were assessed as potential risk factors: age, sex, presence of older/younger siblings, maternal tribe, paternal tribe, location of birth, occupation, frequency of lake contact, type of bathing water, hand-washing behaviour [before eating, after toilet use], footwear outside the house, smoking, alcohol use, helminth infections, exposure to anthelminthic treatment in utero, anthelminthic treatment in last 12 months, maternal and paternal history of allergy/eczema/asthma, BCG scar, immunization history, treatment for malaria in last 12 months, malaria infection, HIV infection and plasma concentration of SEA- and SWA-specific IgE, IgG4 and IgG antibodies.* | | | | | | |

**Table S4. Effect of adjusting for *Sm* infection on associations between non-helminth-related factors and allergy-related outcomes**

| **A. SPT reactivity to *Dermatophagoides mix, B. tropicalis or B. germanica*** | | | | | | | | |
| --- | --- | --- | --- | --- | --- | --- | --- | --- |
|  | | | | | | | | |
|  | **URBAN** | |  | **RURAL** | |  |  | |
| Factor | OR (95% CI) | OR (95% CI) – *Additionally adjusted for Sm (CCA)** |  | OR (95% CI) | OR (95% CI) – *Additionally adjusted for Sm (CCA)** |  | Interaction p | Interaction p** |
|  |  |  |  |  |  |  |  |  |
| Age | **1.02 (1.00, 1.03)** | **1.02 (1.00, 1.03)** |  | **1.02 (1.00, 1.03)** | 1.01 (0.99, 1.03) |  | 0.384 | 0.544 |
|  |  |  |  |  |  |  |  |  |
| Sex |  |  |  |  |  |  |  |  |
| Male | 1 | 1 |  | 1 | 1 |  |  |  |
| Female | 0.71 (0.49, 1.02) | 0.74 (0.52, 1.05) |  | 1.09 (0.79, 1.52) | 0.95 (0.67, 1.35) |  | **0.015** | 0.057 |
| Older siblings (Yes/No) |  |  |  |  |  |  |  |  |
| No | 1 | 1 |  | 1 | 1 |  |  |  |
| Yes | 1.58 (0.90, 2.76) | 1.46 (0.83, 2.58) |  | 0.76 (0.56, 1.03) | **0.69 (0.51, 0.96)** |  | 0.133 | 0.139 |
| Maternal tribe |  |  |  |  |  |  |  |  |
| Central Uganda | 1 | 1 |  | 1 | 1 |  |  |  |
| Other, Ugandan | 0.82 (0.52, 1.30) | 0.79 (0.49, 1.30) |  | 0.86 (0.59, 1.27) | 0.81 (0.53, 1.22) |  |  |  |
| Non-Ugandan | **1.77 (1.17, 2.70)** | **1.83 (1.23, 2.71)** |  | 0.76 (0.44, 1.32) | 0.75 (0.41, 1.36) |  | 0.127 | 0.088 |
| Maternal history of allergies |  |  |  |  |  |  |  |  |
| No | 1 | 1 |  | 1 | 1 |  |  |  |
| Yes | 1.68 (0.89, 3.18) | **2.2 (1.02, 4.96)** |  | 0.90 (0.58, 1.41) | 0.95 (0.59, 1.51) |  | **0.013** | **0.013** |
| Location of birth |  |  |  |  |  |  |  |  |
| City | 1 | 1 |  | 1 | 1 |  |  |  |
| Town | 0.56 (0.30, 1.02) | 0.70 (0.35, 1.38) |  | 0.75 (0.37, 1.52) | 0.82 (0.36, 1.85) |  |  |  |
| Village | **0.34 (0.18, 0.61)** | **0.39 (0.23, 0.67)** |  | 0.61 (0.29, 1.28) | 0.62 (0.28, 1.35) |  | **0.041** | 0.086 |
| BCG scar |  |  |  |  |  |  |  |  |
| No | 1 | 1 |  | 1 | 1 |  |  |  |
| Yes | **2.22 (1.24, 3.97)** | **2.12 (1.17, 3.84)** |  | 1.31 (0.96, 1.79) | 1.35 (0.95, 1.91) |  | 0.601 | 0.863 |
| Handwashing after toilet use |  |  |  |  |  |  |  |  |
| No | 1 | 1 |  | 1 | 1 |  |  |  |
| Yes | 4.67 (0.88, 24.8) | 3.83 (0.73, 20.2) |  | 0.78 (0.59, 1.02) | **0.76 (0.59, 0.96)** |  | **0.001** | **0.005** |
| Malaria treatment, last 12months |  |  |  |  |  |  |  |  |
| No | 1 | 1 |  | 1 | 1 |  |  |  |
| Yes | 0.86 (0.52, 1.42) | 0.91 (0.52, 1.60) |  | 1.08 (0.85, 1.38) | 1.11 (0.87, 1.41) |  | 0.730 | 0.885 |
| HIV |  |  |  |  |  |  |  |  |
| Negative | 1 | 1 |  | 1 | 1 |  |  |  |
| Positive | 1.82 (0.56, 5.93) | 1.86 (0.58, 5.90) |  | 1.17 (0.74, 1.85) | 1.12 (0.71, 1.79) |  | 0.440 | 0.693 |
|  | | | | | | | | |
| **B. IgE sensitisation (ImmunoCAP IgE > 0.35 kU/L) to any of *D. pteronyssinus*, *A. hypogaea* or *B. germanica*** | | | | | | | | |
|  | | | | | | | | |
| Factor | OR (95% CI) | OR (95% CI) – *Additionally adjusted for Sm (CCA)** |  | OR (95% CI) | OR (95% CI) – *Additionally adjusted for Sm (CCA)** |  | Interaction p | Interaction p** |
|  |  |  |  |  |  |  |  |  |
| Age | 0.99 (0.98, 1.01) | 0.99 (0.98, 1.01) |  | 1.01 (0.98, 1.03) | 1.01 (0.98, 1.03) |  | 0.728 | 0.999 |
| Sex |  |  |  |  |  |  |  |  |
| Male | 1 | 1 |  | 1 | 1 |  |  |  |
| Female | 0.77 (0.51, 1.15) | 0.73 (0.52, 1.03) |  | 0.69 (0.42, 1.14) | 0.74 (0.43, 1.29) |  | 0.407 | 0.511 |
| Younger siblings (Yes/No) |  |  |  |  |  |  |  |  |
| No | 1 | 1 |  | 1 | 1 |  |  |  |
| Yes | **2.07 (1.07, 4.01)** | **2.23 (1.17, 4.23)** |  | 0.76 (0.53, 1.09) | 0.72 (0.46, 1.12) |  | **0.008** | **0.002** |
| Handwashing after toilet use |  |  |  |  |  |  |  |  |
| No | 1 | 1 |  | 1 | 1 |  |  |  |
| Yes | 1.36 (0.69, 2.66) | 1.42 (0.67, 2.96) |  | **0.43 (0.30, 0.61)** | **0.41 (0.28, 0.61)** |  | **0.003** | **0.003** |
| Slept under mosquito net last night? |  |  |  |  |  |  |  |  |
| No | 1 | 1 |  | 1 | 1 |  |  |  |
| Yes | 0.93 (0.52, 1.66) | 0.89 (0.48, 1.66) |  | **0.63 (0.41, 0.97)** | **0.61 (0.39, 0.95)** |  | 0.316 | 0.317 |
| Malaria treatment, last 12months |  |  |  |  |  |  |  |  |
| No | 1 | 1 |  | 1 | 1 |  |  |  |
| Yes | 0.78 (0.46, 1.35) | 0.79 (0.43, 1.45) |  | **0.52 (0.34, 0.81)** | **0.55 (0.34, 0.87)** |  | 0.185 | 0.300 |
|  | | | | | | | | |
| **C. Self-reported recent wheeze** | | | | | | | | |
|  | | | | | | | | |
| Factor | OR (95% CI) | OR (95% CI) – *Additionally adjusted for Sm (CCA)** |  | OR (95% CI) | OR (95% CI) – *Additionally adjusted for Sm (CCA)** |  | Interaction p | Interaction p** |
|  |  |  |  |  |  |  |  |  |
| Age | 1.01 (0.99, 1.02) | 1.02 (1.00, 1.03) |  | 1.00 (1.00, 1.03) | 1.03 (1.01, 1.04) |  | 0.386 | 0.378 |
| Sex |  |  |  |  |  |  |  |  |
| Male | 1 | 1 |  | 1 | 1 |  |  |  |
| Female | 1.30 (0.55, 3.07) | 1.55 (0.82, 2.90) |  | **0.47 (0.31, 0.73)** | **0.55 (0.34, 0.89)** |  | 0.087 | 0.057 |
| Older siblings (Yes/No) |  |  |  |  |  |  |  |  |
| No | 1 | 1 |  | 1 | 1 |  |  |  |
| Yes | **0.36 (0.19, 0.73)** | **0.29 (0.11, 0.77)** |  | 0.88 (0.43, 1.79) | 0.91 (0.31, 2.67) |  | 0.177 | 0.108 |
| Maternal history of allergies |  |  |  |  |  |  |  |  |
| No | 1 | 1 |  | 1 | 1 |  |  |  |
| Yes | 3.34 (0.82, 13.6) | 2.62 (0.77, 8.92) |  | 1.11 (0.45, 2.71) | 1.25 (0.47, 3.35) |  | 0.083 | 0.162 |
| Paternal history of allergies |  |  |  |  |  |  |  |  |
| No | 1 | 1 |  | 1 | 1 |  |  |  |
| Yes | 4.96 (0.81, 30.4) | 5.30 (0.77, 36.1) |  | 2.32 (0.97, 5.49) | **3.29 (1.34, 8.10)** |  | 0.259 | 0.437 |
| Paternal tribe |  |  |  |  |  |  |  |  |
| Central Uganda | 1 | 1 |  | 1 | 1 |  |  |  |
| Other, Ugandan | 1.58 (0.35, 7.16) | 1.22 (0.27, 5.48) |  | 0.66 (0.38, 1.18) | 0.62 (0.27, 1.41) |  |  |  |
| Non-Ugandan | **4.25 (1.94, 9.34)** | **5.59 (2.19, 14.2)** |  | 0.61 (0.36, 1.15) | 0.46 (0.19, 1.06) |  | **<0.001** | **<0.001** |
| Handwashing before eating |  |  |  |  |  |  |  |  |
| No | 1 | 1 |  | 1 | 1 |  |  |  |
| Yes | **0.11 (0.04, 0.30)** | **0.15 (0.03, 0.71)** |  | 1.08 (0.25, 4.68) | 0.79 (0.17, 3.68) |  | 0.051 | 0.236 |
|  | | | | | | | | |
| **D. Urticarial rash** | | | | | | | | |
|  | | | | | | | | |
| Factor | OR (95% CI) | OR (95% CI) – *Additionally adjusted for Sm (CCA)** |  | OR (95% CI) | OR (95% CI) – *Additionally adjusted for Sm (CCA)** |  | Interaction p | Interaction p** |
|  |  |  |  |  |  |  |  |  |
| Age | 1.02 (0.99, 1.04) | 1.01 (0.99, 1.04) |  | 1.03 (1.02, 1.03) | 1.03 (1.02, 1.04) |  | 0.512 | 0.501 |
| Sex |  |  |  |  |  |  |  |  |
| Male | 1 | 1 |  | 1 | 1 |  |  |  |
| Female | 0.98 (0.54, 1.79) | 1.07 (0.55, 2.10) |  | 1.12 (0.85, 1.48) | 1.23 (0.84, 1.80) |  | 0.398 | 0.292 |
| Maternal tribe |  |  |  |  |  |  |  |  |
| Central Uganda | 1 | 1 |  | 1 | 1 |  |  |  |
| Other, Ugandan | 0.77 (0.40, 1.49) | 0.80 (0.39, 1.65) |  | 1.58 (0.98, 2.54) | 1.65 (0.97, 2.79) |  |  |  |
| Non-Ugandan | 1.18 (0.35, 4.03) | 1.15 (0.29, 4.55) |  | 1.74 (0.97, 3.11) | **2.07 (1.16, 3.69)** |  | 0.970 | 0.997 |
| Paternal tribe |  |  |  |  |  |  |  |  |
| Central Uganda | 1 | 1 |  | 1 | 1 |  |  |  |
| Other, Ugandan | 1.08 (0.55, 3.28) | 1.03 (0.42, 2.52) |  | **0.43 (0.29, 0.65)** | **0.43 (0.27, 0.68)** |  |  |  |
| Non-Ugandan | 1.44 (0.63, 3.28) | 1.28 (0.34, 4.80) |  | 0.62 (0.35, 1.08) | 0.44 (0.23, 0.84) |  | 0.193 | 0.411 |
| Maternal history of allergies |  |  |  |  |  |  |  |  |
| No | 1 | 1 |  | 1 | 1 |  |  |  |
| Yes | 2.29 (0.83, 6.32) | 2.43 (0.84, 7.03) |  | **2.19 (1.50, 3.21)** | **2.23 (1.51, 3.30)** |  | 0.685 | 0.513 |
| Paternal history of allergies |  |  |  |  |  |  |  |  |
| No | 1 | 1 |  | 1 | 1 |  |  |  |
| Yes | 2.19 (0.84, 5.70) | 1.62 (0.68, 3.87) |  | 0.79 (0.39, 1.59) | 0.90 (0.46, 1.76) |  | 0.096 | 0.122 |
| Malaria treatment, last 12 months |  |  |  |  |  |  |  |  |
| No | 1 | 1 |  | 1 | 1 |  |  |  |
| Yes | 1.25 (0.78, 2.01) | 1.21 (0.71, 2.07) |  | **1.69 (1.25, 2.30)** | **1.69 (1.14, 2.51)** |  | 0.149 | 0.165 |
| HIV |  |  |  |  |  |  |  |  |
| Negative | 1 | 1 |  | 1 | 1 |  |  |  |
| Positive | 3.19 (0.80, 12.7) | 2.67 (0.64, 11.1) |  | 1.05 (0.63, 1.77) | 1.02 (0.62, 1.69) |  | 0.595 | 0.391 |
|  | | | | | | | | |
| **E. Rhinitis** | | | | | | | | |
|  | | | | | | | | |
| Factor | OR (95% CI) | OR (95% CI) – *Additionally adjusted for Sm (CCA)** |  | OR (95% CI) | OR (95% CI) – *Additionally adjusted for Sm (CCA)** |  | Interaction p | Interaction p** |
|  |  |  |  |  |  |  |  |  |
| Age | 0.99 (0.97, 1.01) | 0.99 (0.97, 1.01) |  | 1.02 (1.00, 1.03) | 1.02 (1.00, 1.04) |  | 0.576 | 0.597 |
| Sex |  |  |  |  |  |  |  |  |
| Male | 1 | 1 |  | 1 | 1 |  |  |  |
| Female | 1.31 (0.71, 2.40) | 1.38 (0.69, 2.73) |  | 1.42 (0.69, 2.88) | 1.48 (0.72, 3.05) |  | 0.786 | 0.212 |
| Older siblings (Yes/No) |  |  |  |  |  |  |  |  |
| No | 1 | 1 |  | 1 | 1 |  |  |  |
| Yes | 1.72 (0.73, 4.08) | 1.67 (0.71, 3.96) |  | **0.63 (0.46, 0.86)** | **0.61 (0.42, 0.88)** |  | 0.098 | **0.040** |
| Younger siblings (Yes/No) |  |  |  |  |  |  |  |  |
| No | 1 | 1 |  | 1 | 1 |  |  |  |
| Yes | 1.59 (0.80, 3.15) | 1.58 (0.83, 2.99) |  | 2.07 (0.67, 6.41) | 1.81 (0.59, 5.53) |  | 0.859 | 0.740 |
| Location of birth |  |  |  |  |  |  |  |  |
| City | 1 | 1 |  | 1 | 1 |  |  |  |
| Town | 0.09 (0.02, 5.55) | 0.08 (0.00, 2.48) |  | **0.50 (0.29, 0.85)** | 0.66 (0.29, 1.49) |  |  |  |
| Village | 0.29 (0.03, 3.05) | 0.22 (0.03, 1.68) |  | **0.28 (0.12, 0.64)** | 0.42 (0.15, 1.19) |  | 0.135 | 0.241 |
| Maternal history of allergies |  |  |  |  |  |  |  |  |
| No | 1 | 1 |  | 1 | 1 |  |  |  |
| Yes | **3.56 (1.28, 9.93)** | **3.95 (1.41, 11.1)** |  | 1.58 (0.85, 2.96) | 1.69 (0.96, 2.97) |  | 0.676 | 0.878 |
| Paternal history of allergies |  |  |  |  |  |  |  |  |
| No | 1 | 1 |  | 1 | 1 |  |  |  |
| Yes | **3.29 (1.24, 8.76)** | **3.56 (1.28, 9.93)** |  | 1.30 (0.61, 2.76) | 1.15 (0.48, 2.79) |  | 0.435 | 0.264 |
| Hand washing after toilet |  |  |  |  |  |  |  |  |
| No | 1 | 1 |  | 1 | 1 |  |  |  |
| Yes | 2.26 (0.68, 7.47) | 2.54 (0.81, 7.95) |  | 1.91 (0.92, 3.94) | 1.91 (0.85, 4.32) |  | 0.212 | 0.221 |
| HIV |  |  |  |  |  |  |  |  |
| Negative | 1 | 1 |  | 1 | 1 |  |  |  |
| Positive | 3.15 (1.18, 8.39) | 2.48 (0.59, 10.4) |  | 1.17 (0.57, 2.40) | 1.34 (0.66, 2.72) |  | 0.029 | 0.228 |
|  | | | | | | | | |
| *Table shows only non-helminth-related factors that were associated with SPT reactivity, asIgE sensitisation, wheeze, urticaria and rhinitis in either the urban or the rural survey. All other non-helminth-related factors that were assessed are listed in the statistical methods section. Significant associations are highlighted in bold.*  ** Adjusting for other Sm infection variables (KK / PCR), Sm infection intensity (KK) and SWA- and SEA-specific antibodies paints a similar picture.*  *** Additionally adjusted for Sm infection (CCA)*  *CCA: Circulating Cathodic Antigen.* | | | | | | | | |

**Table S5. Crude and adjusted associations with IgE sensitisation (ImmunoCAP IgE > 0.35 kU/L) to any of *D. pteronyssinus*, *A. hypogaea* or *B. germanica***

|  |  | UNADJUSTED ANALYSIS | | | | | |  | ADJUSTED ANALYSIS | | | | |
| --- | --- | --- | --- | --- | --- | --- | --- | --- | --- | --- | --- | --- | --- |
|  |  |  |  | |  | | |  |  | | | | |
|  |  |  | **URBAN** |  |  | **RURAL** |  |  | **URBAN** |  | **RURAL** |  |  |
| Factor |  | N (%)^≠^ | OR (95% CI)¶ | p | N (%)^≠^ | OR (95% CI)¶ | p |  | OR (95% CI) ¶# | p | OR (95% CI)¶§ | p | Interaction p |
|  |  |  |  |  |  |  |  |  |  |  |  |  |  |
| Age |  |  | 0.99 (0.98, 1.01) | 0.400 |  | 1.00 (0.99, 1.02) | 0.076 |  | 0.99 (0.98, 1.01) | 0.547 | 1.01 (0.98, 1.03) | 0.589 | 0.728 |
| Sex |  |  |  |  |  |  |  |  |  |  |  |  |  |
| Male |  | 47 (48) | 1 |  | 241 (64) | 1 |  |  | 1 |  | 1 |  |  |
| Female |  | 101 (41) | 0.75 (0.50, 1.10) | 0.136 | 196 (49) | **0.56 (0.36, 0.86)** | **0.011** |  | 0.77 (0.51, 1.15) | 0.200 | 0.69 (0.42, 1.14) | 0.140 | 0.407 |
| Younger siblings (Yes/No) |  |  |  |  |  |  |  |  |  |  |  |  |  |
| No |  | 27 (33) | 1 |  | 61 (62) | 1 |  |  | 1 |  | 1 |  |  |
| Yes |  | 106 (46) | 1.76 (0.96, 3.25) | 0.065 | 313 (56) | 0.81 (0.57, 1.14) | 0.213 |  | **2.07 (1.07, 4.01)** | **0.030** | 0.76 (0.53, 1.09) | 0.129 | **0.008** |
| Occupation |  |  |  |  |  |  |  |  |  |  |  |  |  |
| Student or child (not at school) |  | 68 (48) | 1 |  | 64 (51) | 1 |  |  | 1 |  | 1 |  |  |
| Unemployed or housewife |  | 34 (40) | 0.70 (0.42, 1.16) |  | 58 (54) | 1.23 (0.75, 2.01) |  |  | 0.70 (0.31, 1.60) |  | **2.05 (1.38, 3.03)** |  |  |
| Agricultural, fishing or lake related |  | 4 (36) | 0.61 (0.17, 2.12) |  | 251 (61) | **1.58 (1.12, 2.24)** |  |  | 0.56 (0.13, 2.46) |  | **1.87 (1.04, 3.37)** |  |  |
| Professional or service providers |  | 27 (38) | 0.64 (0.37, 1.11) | 0.391 | 61 (47) | 0.78 (0.49, 1.25) | **0.020** |  | 0.61 (0.26, 1.43) | 0.725 | 1.38 (0.72, 2.66) | **0.014** | 0.148 |
| Lake contact |  |  |  |  |  |  |  |  |  |  |  |  |  |
| Never |  | 39 (45) | 1 |  |  |  |  |  | 1 |  |  |  |  |
| Rarely |  | 69 (42) | 0.89 (0.49, 1.62) |  | 8 (42) | 1 |  |  | 0.98 (0.52, 1.85) |  | 1 |  |  |
| Once a month |  | 11 (35) | 0.67 (0.36, 1.26) |  |  |  |  |  | 0.74 (0.38, 1.41) |  |  |  |  |
| Once a week |  | 14 (50) | 1.23 (0.47, 3.19) | 1.000 | 25 (44) | 0.93 (0.29, 3.00) |  |  | 1.32 (0.49, 3.55) | 0.835 | 0.82 (0.23, 2.89) |  |  |
| Daily/ almost daily |  |  |  |  | 343 (59) | 2.22 (0.63, 7.86) | **0.034** |  |  |  | 1.64 (0.45, 5.90) | 0.174 |  |
| Bathe in water from lake? |  |  |  |  |  |  |  |  |  |  |  |  |  |
| No |  | 121 (42) | 1 |  | 15 (63) | 1 |  |  | 1 |  | 1 |  |  |
| Yes |  | 12 (57) | 1.85 (0.51, 6.66) | 0.327 | 361 (57) | 0.49 (0.19, 1.27) | 0.139 |  | 1.86 (0.50, 6.87) | 0.331 | 0.42 (0.15, 1.11) | 0.078 | 0.065 |
| Hand washing after toilet |  |  |  |  |  |  |  |  |  |  |  |  |  |
| No |  | 15 (39) | 1 |  | 148 (71) | 1 |  |  | 1 |  | 1 |  |  |
| Yes |  | 117 (43) | 1.16 (0.61, 2.23) | 0.627 | 228 (50) | **0.37 (0.25, 0.55)** | **<0.001** |  | 1.36 (0.69, 2.66) | 0.344 | **0.43 (0.30, 0.61)** | **<0.001** | **0.003** |
|  |  |  |  |  |  |  |  |  |  |  |  |  |  |
| SWA-specific IgE* |  |  | 2.54 (0.56, 11.4) | 0.209 |  | **4.15 (2.05, 8.44)** | **<0.001** |  | 2.95 (0.51, 17.2) | 0.214 | **6.17 (2.79, 13.6)** | **<0.001** | 0.459 |
| SWA-specific IgG4* |  |  | 1.01 (0.86, 1.18) | 0.898 |  | **1.22 (1.02, 1.45)** | **0.032** |  | 1.01 (0.85, 1.19) | 0.932 | 1.07 (0.89, 1.27) | 0.470 | 0.433 |
| SEA-specific IgG4* |  |  | 1.10 (0.98, 1.24) | 0.090 |  | **1.14 (1.02, 1.26)** | **0.023** |  | 1.11 (0.97, 1.25) | 0.102 | 1.07 (0.95, 1.21) | 0.227 | 0.704 |
| SWA-specific IgG* |  |  | 2.74 (0.99, 7.59) | **0.052** |  | **3.04 (1.82, 5.08)** | **<0.001** |  | **3.33 (1.12, 9.86)** | **0.031** | 1.53 (0.82, 2.86) | 0.177 | 0.340 |
| SEA-specific IgG* |  |  | 1.58 (0.63, 4.01) | 0.310 |  | **2.41 (1.57, 3.66)** | **<0.001** |  | 1.77 (0.63, 4.96) | 0.260 | 1.43 (0.88, 2.30) | 0.138 | 0.796 |
|  |  |  |  |  |  |  |  |  |  |  |  |  |  |
| S. mansoni infection (KK) |  |  |  |  |  |  |  |  |  |  |  |  |  |
| Uninfected |  | 119 (44) | 1 |  | 271 (55) | 1 |  |  | 1 |  | 1 |  |  |
| Infected |  | 6 (43) | 0.95 (0.29, 3.09) | 0.931 | 118 (63) | **1.39 (1.02, 1.90)** | **0.036** |  | 1.06 (0.34, 3.35) | 0.910 | **1.52 (1.19, 1.94)** | **0.002** | 0.180 |
| S. mansoni infection intensity (KK) |  |  |  |  |  |  |  |  |  |  |  |  |  |
| Uninfected |  | 119 (44) | 1 |  | 271 (55) | 1 |  |  | 1 |  | 1 |  |  |
| Light |  | 2 (29) | 0.51 (0.08, 3.09) |  | 54 (57) | 1.23 (0.85, 1.78) |  |  | 0.66 (0.11, 4.04) |  | **1.74 (1.18, 2.54)** |  |  |
| Moderate |  | 2 (50) | 1.26 (0.15, 10.8) |  | 35 (66) | 1.23 (0.65, 2.32) |  |  | 1.20 (0.16, 8.79) |  | 0.94 (0.46, 1.90) |  |  |
| Heavy |  | 2 (67) | 2.54 (0.13, 49.7) | 0.711 | 29 (73) | 2.44 (0.93, 6.38) | **0.019** |  | 2.32 (0.13, 40.9) | 0.662 | 2.37 (0.71, 7.83) | **0.028** | 0.536 |
| Any nematode infection** |  |  |  |  |  |  |  |  |  |  |  |  |  |
| No |  | 109 (42) | 1 |  | 281 (54) | 1 |  |  | 1 |  | 1 |  |  |
| Yes |  | 16 (62) | 2.22 (0.83, 5.94) | 0.108 | 108 (66) | **1.90 (1.29, 2.81)** | **0.002** |  | 2.34 (0.76, 7.19) | 0.130 | 1.53 (0.94, 2.49) | 0.084 | 0.287 |
| Slept under mosquito net last night? |  |  |  |  |  |  |  |  |  |  |  |  |  |
| No |  | 35 (45) | 1 |  | 203 (62) | 1 |  |  | 1 |  | 1 |  |  |
| Yes |  | 97 (42) | 0.88 (0.49, 1.59) | 0.678 | 172 (52) | **0.62 (0.41, 0.94)** | **0.025** |  | 0.93 (0.52, 1.66) | 0.958 | **0.63 (0.41, 0.97)** | **0.037** | 0.316 |
| Malaria treatment, last 12months |  |  |  |  |  |  |  |  |  |  |  |  |  |
| No |  | 83 (45) | 1 |  | 202 (63) | 1 |  |  | 1 |  | 1 |  |  |
| Yes |  | 48 (39) | 0.78 (0.45, 1.34) | 0.352 | 221 (51) | **0.53 (0.36, 0.75)** | **0.001** |  | 0.78 (0.46, 1.35) | 0.365 | **0.52 (0.34, 0.81)** | **0.005** | 0.185 |
|  | | | | | | | | | | | | | |
| *Table shows only factors that were associated with IgE sensitisation (before and/or after adjustment) in either the urban or the rural survey. All other factors that were assessed are listed in the methods section. Significant associations are highlighted in bold. Interaction p values are shown to denote whether tests for interaction, using the adjusted model, showed statistical evidence for urban-rural differences in associations with IgE sensitisation, or not.*  *≠Number (percentage in parenthesis) of IgE sensitised individuals in each category*  *¶Odds ratios (ORs) and 95% confidence intervals (CI) adjusted for survey design*  *§All ORs were adjusted for hand washing after toilet use, mosquito net use, malaria treatment, age and sex.*  *#All ORs were adjusted for age and sex.*  **Log_10_ (concentration+1) transformation applied before analysis*  ***Infection with any of Ascaris lumbricoides, Trichuris trichiura (assessed by KK), Necator americanus, Strongyloides stercoralis (assessed by PCR).*  *KK: Kato-Katz; SWA: Schistosoma adult worm antigen; SEA: Schistosoma egg antigen.* | | | | | | | | | | | | | |

**Table S6. Summary of risk factors for IgE sensitisation (ImmunoCAP IgE > 0.35 kU/L) to individual allergen extracts**

|  |  | **URBAN SURVEY** | |  | **RURAL SURVEY** | |
| --- | --- | --- | --- | --- | --- | --- |
|  |  |  |  |  |  |  |
| **Outcome** |  | **Factors positively associated with outcome** | **Factors inversely associated with outcome** |  | **Factors positively associated with outcome** | **Factors inversely associated with outcome** |
|  |  |  |  |  |  |  |
| *D. pteronyssinus*-specific IgE sensitisation (ImmunoCAP) |  | - Presence of younger siblings (p=0.020) - Paternal history of allergies (p=0.041) - Maternal history of allergies (p=0.034) | - |  | - *T. trichiura* infection (p<0.001) - SWA-specific IgG (p=0.016) - SEA-specific IgG (p=0.037) - Maternal history of allergies (p=0.004) - Age (p=0.036) - engaging in agricultural / fishing / lake related activities or being unemployed (p=0.043) | - Hand washing after toilet use (p=0.008) - Bathing in lake water (p<0.001) - Presence of younger siblings (p=0.008) - Female gender (p=0.001) - Mosquito net use (p=0.052) |
|  |  |  |  |  |  |  |
| *B. germanica*-specific IgE sensitisation (ImmunoCAP) |  | - SEA-specific IgE (p=0.034) - Presence of younger siblings (p=0.045) | - *S. mansoni* infection (CCA) [p=0.024] |  | - SWA-specific IgE (p<0.001) - Any nematode infection (p=0.063) - *Sm* infection (KK) [p=0.060] | - Hand washing after toilet use (p=0.001) - Bathing in lake water (p=0.01) - Malaria infection (p=0.01) - Recent malaria treatment (p=0.010) |
|  |  |  |  |  |  |  |
| *A. hypogaea*-specific IgE sensitisation (ImmunoCAP) |  | - SEA-specific IgE (p=0.051) - Hookworm infection (p=0.031) | - |  | - SEA-specific IgE (p<0.001) - BCG scar (p=0.013) - Worm treatment during pregnancy (p=0.002) | - Recent malaria treatment (p=0.004) |
|  |  |  |  |  |  |  |
| *D. pteronyssinus*-specific IgE (ImmunoCAP) – continuous variable |  | - | - |  | - SWA-specific IgE (p=0.004), IgG (p=0.027) - SEA-specific IgG (p=0.035) - *S. mansoni* infection (KK) (p=0.029) - Paternal history of allergies (p=0.028) | - Female gender (p<0.001) - Recent malaria treatment (p=0.029) - Bathing in lake water (p=0.023) |
|  |  |  |  |  |  |  |
| *B. germanica*-specific IgE sensitisation (ImmunoCAP)- continuous variable |  | - SWA-specific IgG (p=0.011) - SEA-specific IgE (p=0.001) | - |  | - SWA-specific IgE (p<0.001) and IgG (p=0.008) - SEA-specific IgG (p=0.027) - *A. lumbricoides* infection (p<0.001) | - Hand washing (before eating [p=0.040], after toilet [p=0.002]) - Bathing in lake water (p=0.008) - Malaria infection (p<0.001) - Recent malaria treatment (p=0.005) - Female gender (p=0.004), |
|  |  |  |  |  |  |  |
| *A. hypogaea*-specific IgE sensitisation (ImmunoCAP)- continuous variable |  | - *S. mansoni* infection [PCR] (p=0.008) - *S. stercoralis* infection (p<0.001) - Hookworm infection (p=0.003) - Any nematode infection (p=0.002) - SEA-specific IgE (p=0.002), IgG4 (p<0.001) and IgG (p=0.025) and IgG4 (p=0.001) - SWA-specific IgG (p=0.003) - Presence of younger siblings (p=0.034) | - |  | - SWA-specific IgE (p<0.001), IgG (p<0.001), - SEA-specific IgE (p=0.001), IgG (p<0.001), - Hookworm infection (p=0.017) | - Female gender (p=0.011) - Recent malaria treatment (p=0.002) - Bathing in lake water (p=0.005) - Malaria infection (p=0.043) - Hand washing after toilet use (p=0.028) |
|  |  |  |  |  |  |  |
| *P values are from adjusted analyses, conducted as described in the main text. The following were assessed as potential risk factors: age, sex, presence of older/younger siblings, maternal tribe, paternal tribe, location of birth, occupation, frequency of lake contact, type of bathing water, hand-washing behaviour [before eating, after toilet use], footwear outside the house, smoking, alcohol use, helminth infections, exposure to anthelminthic treatment in utero, anthelminthic treatment in last 12 months, maternal and paternal history of allergy/eczema/asthma, BCG scar, immunization history, treatment for malaria in last 12 months, malaria infection, HIV infection and plasma concentration of SEA- and SWA-specific IgE, IgG4 and IgG antibodies.* | | | | | | |

**Table S7. Crude and adjusted associations with clinical allergy-related outcomes**

|  |  |  |  | | UNADJUSTED ANALYSIS | | |  | ADJUSTED ANALYSIS | | | | |
| --- | --- | --- | --- | --- | --- | --- | --- | --- | --- | --- | --- | --- | --- |
|  |  |  |  | |  | | |  |  | | | | |
|  |  |  | URBAN |  |  | RURAL |  |  | URBAN |  | RURAL | |  |
| **WHEEZE (AGE≥ 5 YEARS)** | | | | | | | | | | | | | |
|  |  | N (%)^≠^ | OR (95% CI)^a^ | p | N (%)^≠^ | OR (95% CI)^a^ | p |  | OR (95% CI)^a,b^ | p | OR (95% CI)^a,c^ | p | Interaction p |
|  |  |  |  |  |  |  |  |  |  |  |  |  |  |
| Age |  |  | 1.02 (0.99, 1.03) | 0.067 |  | **1.02 (1.01, 1.04)** | **0.001** |  | 1.01 (0.99, 1.02) | 0.240 | **1.00 (1.00, 1.03)** | **0.003** | 0.386 |
| Sex |  |  |  |  |  |  |  |  |  |  |  |  |  |
| Male |  | 8 (2) | 1 |  | 56 (4) | 1 |  |  | 1 |  | 1 |  |  |
| Female |  | 16 (2) | 1.19 (0.59, 2.42) | 0.602 | 31 (2) | **0.45 (0.29, 0.69)** | **0.001** |  | 1.30 (0.55, 3.07) | 0.531 | **0.47 (0.31, 0.73)** | **0.002** | 0.087 |
| Older siblings (Yes/No) |  |  |  |  |  |  |  |  |  |  |  |  |  |
| No |  | 11 (3) | 1 |  | 20 (4) | 1 |  |  | 1 |  | 1 |  |  |
| Yes |  | 13 (2) | **0.46 (0.24, 0.89)** | **0.023** | 62 (4) | 0.84 (0.39, 1.77) | 0.642 |  | **0.36 (0.19, 0.73)** | **0.006** | 0.88 (0.43, 1.79) | 0.706 | 0.177 |
| Occupation |  |  |  |  |  |  |  |  |  |  |  |  |  |
| Student or child (not at school) |  | 12 (3) | 1 |  | 6 (1) | 1 |  |  | 1 |  | 1 |  |  |
| Unemployed or housewife |  | 7 (2) | 0.88 (0.51, 1.54) |  | 8 (3) | 1.24 (0.36, 4.22) |  |  | 0.46 (0.15, 1.41) |  | 1.68 (0.45, 6.31) |  |  |
| Agricultural, fishing or lake related |  | 2 (3) | 1.26 (0.32, 4.94) |  | 61 (4) | **4.35 (1.38, 13.6)** |  |  | 0.44 (0.03, 6.96) |  | 3.85 (0.96, 15.4) |  |  |
| Professional or service providers |  | 3 (1) | 0.35 (0.07, 1.55) | 0.563 | 12 (3) | 3.10 (0.86, 11.1) | **0.035** |  | **0.08 (0.01, 0.77)** | 0.194 | 3.75 (0.79, 17.6) | 0.258 | 0.051 |
| Maternal history of allergies |  |  |  |  |  |  |  |  |  |  |  |  |  |
| No |  | 15 (2) | 1 |  | 59 (3) | 1 |  |  | 1 |  | 1 |  |  |
| Yes |  | 7 (7) | **4.18 (1.65, 10.6)** | **0.004** | 11 (3) | 0.95 (0.38, 2.38) | 0.926 |  | 3.34 (0.82, 13.6) | 0.089 | 1.11 (0.45, 2.71) | 0.818 | 0.083 |
| Paternal history of allergies |  |  |  |  |  |  |  |  |  |  |  |  |  |
| No |  | 14 (2) | 1 |  | 55 (3) | 1 |  |  | 1 |  | 1 |  |  |
| Yes |  | 5 (9) | **5.73 (1.34, 24.5)** | **0.021** | 9 (4) | 2.11 (0.96, 4.61) | 0.061 |  | 4.96 (0.81, 30.4) | 0.080 | **2.32 (0.97, 5.49)** | **0.056** | 0.259 |
| Paternal tribe |  |  |  |  |  |  |  |  |  |  |  |  |  |
| Central Uganda |  | 8 (2) | 1 |  | 38 (3) | 1 |  |  | 1 |  | 1 |  |  |
| Other, Ugandan |  | 12 (2) | 1.41 (0.50, 3.96) |  | 39 (3) | 0.68 (0.38, 1.20) |  |  | 1.58 (0.35, 7.16) |  | 0.66 (0.38, 1.18) |  |  |
| Non-Ugandan, African |  | 4 (4) | **2.66 (1.14, 6.19)** | 0.061 | 9 (3) | 0.65 (0.36, 1.15) | 0.193 |  | **4.25 (1.94, 9.34)** | **0.001** | 0.61 (0.36, 1.15) | 0.173 | **<0.001** |
| Hand washing before eating |  |  |  |  |  |  |  |  |  |  |  |  |  |
| No |  | 1 (1) | 1 |  | 3 (3) | 1 |  |  | 1 |  | 1 |  |  |
| Yes |  | 23 (2) | **0.17 (0.05, 0.54)** | **0.004** | 79 (4) | 0.82 (0.19, 3.51) | 0.785 |  | **0.11 (0.04, 0.30)** | **<0.001** | 1.08 (0.25, 4.68) | 0.914 | 0.051 |
|  |  |  |  |  |  |  |  |  |  |  |  |  |  |
| SWA-specific IgG* |  |  | 0.21 (0.02, 2.18) | 0.183 |  | **27.4 (4.6, 162.3)** | **0.001** |  | **0.13 (0.02, 0.81)** | **0.030** | **26.8 (4.6, 158.6)** | **0.001** | **<0.001** |
| SEA-specific IgG* |  |  | **0.24 (0.06, 0.97)** | **0.046** |  | **4.87 (1.09, 21.6)** | **0.038** |  | **0.15 (0.05, 0.54)** | **0.005** | **5.91 (1.07, 32.4)** | **0.041** | **0.001** |
| SWA-specific IgG4* |  |  | **1.28 (1.02, 1.63)** | **0.037** |  | 4.53 (0.84, 24.4) | 0.076 |  | 1.46 (0.98, 2.19) | 0.060 | 5.38 (0.73, 39.9) | 0.096 | 0.276 |
|  |  |  |  |  |  |  |  |  |  |  |  |  |  |
| Any nematode infection |  |  |  |  |  |  |  |  |  |  |  |  |  |
| No |  | 16 (2) | 1 |  | 60 (4) | 1 |  |  | 1 |  | 1 |  |  |
| Yes |  | 1 (1) | 0.51 (0.06, 4.22) | 0.513 | 12 (2) | 0.45 (0.19, 1.05) | 0.064 |  | 0.92 (0.10, 8.27) | 0.937 | **0.41 (0.18, 0.97)** | **0.042** | 0.744 |
|  |  |  |  |  |  |  |  |  |  |  |  |  |  |
| **URTICARIAL RASH** | | | | | | | | | | | | | |
|  |  | N (%)^≠^ | OR (95% CI)^a^ | p | N (%)^≠^ | OR (95% CI)^a^ | p |  | OR (95% CI)^a,d^ | p | OR (95% CI)^a,e^ | p | Interaction p |
|  |  |  |  |  |  |  |  |  |  |  |  |  |  |
| Age |  |  | 1.02 (0.99, 1.04) | 0.059 |  | **1.03 (1.02, 1.04)** | **<0.001** |  | 1.02 (0.99, 1.04) | 0.179 | **1.03 (1.02, 1.03)** | **<0.001** | 0.512 |
| Sex |  |  |  |  |  |  |  |  |  |  |  |  |  |
| Male |  | 20 (4) | 1 |  | 160 (9) | 1 |  |  | 1 |  | 1 |  |  |
| Female |  | 33 (4) | 1.09 (0.64, 1.88) | 0.734 | 174 (11) | 1.21 (0.94, 1.54) | 0.132 |  | 0.98 (0.54, 1.79) | 0.956 | 1.12 (0.85, 1.48) | 0.399 | 0.398 |
| Occupation |  |  |  |  |  |  |  |  |  |  |  |  |  |
| Student or child (not at school) |  | 19 (3) | 1 |  | 74 (6) | 1 |  |  | 1 |  | 1 |  |  |
| Unemployed or housewife |  | 13 (4) | 1.57 (0.63, 3.91) |  | 45 (15) | **2.92 (1.86, 4.56)** |  |  | 0.74 (0.36, 1.52) |  | 1.49 (0.73, 3.00) |  |  |
| Agricultural, fishing or lake related |  | 5 (8) | 3.13 (0.93, 10.5) |  | 162 (12) | **2.45 (1.68, 3.56)** |  |  | 0.43 (0.04, 4.56) |  | 1.25 (0.68, 2.28) |  |  |
| Professional or service providers |  | 16 (5) | 1.76 (0.85, 3.63) | 0.154 | 53 (11) | **2.14 (1.49, 3.06)** | **<0.001** |  | 1.25 (0.33, 4.76) | 0.723 | 0.91 (0.48, 1.70) | 0.674 | 0.595 |
| Maternal tribe |  |  |  |  |  |  |  |  |  |  |  |  |  |
| Central Uganda |  | 22 (4) | 1 |  | 100 (8) | 1 |  |  | 1 |  | 1 |  |  |
| Other, Ugandan |  | 23 (4) | 1.04 (0.59, 1.84) |  | 158 (10) | 1.22 (0.84, 1.78) |  |  | 0.77 (0.40, 1.49) |  | 1.58 (0.98, 2.54) |  |  |
| Non-Ugandan, African |  | 8 (8) | 1.92 (0.85, 4.32) | 0.254 | 72 (14) | **1.61 (1.17, 2.22)** | **0.017** |  | 1.18 (0.35, 4.03) | 0.737 | 1.74 (0.97, 3.11) | 0.091 | 0.970 |
| Paternal tribe |  |  |  |  |  |  |  |  |  |  |  |  |  |
| Central Uganda |  | 22 (4) | 1 |  | 158 (12) | 1 |  |  | 1 |  | 1 |  |  |
| Other, Ugandan |  | 25 (4) | 1.08 (0.54, 2.18) |  | 127 (8) | **0.69 (0.55, 0.88)** |  |  | 1.08 (0.55, 3.28) |  | **0.43 (0.29, 0.65)** |  |  |
| Non-Ugandan, African |  | 6 (5) | 1.41 (0.65, 3.04) | 0.644 | 49 (12) | 0.98 (0.71, 1.37) | **0.013** |  | 1.44 (0.63, 3.28) | 0.625 | 0.62 (0.35, 1.08) | **0.001** | 0.193 |
| Maternal history of allergies |  |  |  |  |  |  |  |  |  |  |  |  |  |
| No |  | 36 (3) | 1 |  | 206 (9) | 1 |  |  | 1 |  | 1 |  |  |
| Yes |  | 11 (9) | **2.68 (1.26, 5.72)** | **0.013** | 70 (13) | **1.82 (1.31, 2.52)** | **0.001** |  | 2.29 (0.83, 6.32) | 0.103 | **2.19 (1.50, 3.21)** | **<0.001** | 0.566 |
| Paternal history of allergies |  |  |  |  |  |  |  |  |  |  |  |  |  |
| No |  | 36 (3) | 1 |  | 235 (9) | 1 |  |  | 1 |  | 1 |  |  |
| Yes |  | 6 (9) | **2.72 (1.05, 6.98)** | **0.039** | 31 (10) | 0.97 (0.52, 1.83) | 0.946 |  | 2.19 (0.84, 5.70) | 0.102 | 0.79 (0.39, 1.59) | 0.899 | 0.096 |
|  |  |  |  |  |  |  |  |  |  |  |  |  |  |
| SEA-specific IgE* |  |  | 0.89 (0.55, 1.44) | 0.630 |  | 2.13 (0.81, 5.61) | 0.122 |  | 0.72 (0.36, 1.46) | 0.348 | **2.83 (1.44, 5.55)** | **0.004** | **0.022** |
|  |  |  |  |  |  |  |  |  |  |  |  |  |  |
| Bathe in water from lake? |  |  |  |  |  |  |  |  |  |  |  |  |  |
| No |  | 47 (4) | 1 |  | 6 (8) | 1 |  |  | 1 |  | 1 |  |  |
| Yes |  | 6 (5) | 1.40 (0.67, 2.89) | 0.343 | 264 (12) | **3.03 (1.26, 7.24)** | **0.015** |  | 0.87 (0.32, 2.37) | 0.776 | 3.44 (0.09, 118) | 0.479 | 0.147 |
| Worm treatment, last 12 months |  |  |  |  |  |  |  |  |  |  |  |  |  |
| No |  | 13 (3) | 1 |  | 18 (5) | 1 |  |  | 1 |  | 1 |  |  |
| Yes |  | 40 (5) | **1.98 (1.17, 3.35)** | **0.013** | 316 (11) | **2.04 (1.40, 2.96)** | **0.001** |  | **2.11 (1.11, 4.01)** | **0.024** | 1.40 (0.71, 2.77) | 0.171 | 0.248 |
| Worm treatment in pregnancy |  |  |  |  |  |  |  |  |  |  |  |  |  |
| No |  | 8 (4) | 1 |  | 11 (11) | 1 |  |  | 1 |  | 1 |  |  |
| Yes |  | 16 (3) | 0.71 (0.22, 2.24) | 0.542 | 6 (5) | **0.25 (0.07, 0.86)** | **0.030** |  | 1.02 (0.32, 3.26) | 0.975 | 0.29 (0.07, 1.14) | 0.074 | 0.508 |
| Malaria treatment, last 12 months |  |  |  |  |  |  |  |  |  |  |  |  |  |
| No |  | 29 (4) | 1 |  | 90 (7) | 1 |  |  | 1 |  | 1 |  |  |
| Yes |  | 23 (5) | 1.28 (0.81, 2.05) | 0.278 | 240 (12) | **1.68 (1.22, 2.32)** | **0.002** |  | 1.25 (0.78, 2.01) | 0.329 | **1.69 (1.25, 2.30)** | **0.001** | 0.149 |
| HIV |  |  |  |  |  |  |  |  |  |  |  |  |  |
| Negative |  | 43 (4) | 1 |  | 210 (11) | 1 |  |  | 1 |  | 1 |  |  |
| Positive |  | 5 (9) | 2.49 (0.92, 6.79) | 0.071 | 65 (16) | **1.55 (1.07, 2.22)** | **0.020** |  | 3.19 (0.80, 12.7) | 0.095 | 1.05 (0.63, 1.77) | 0.829 | 0.595 |
|  |  |  |  |  |  |  |  |  |  |  |  |  |  |
| **RHINITIS** |  |  |  |  |  |  |  |  |  |  |  |  |  |
|  |  | N (%)^≠^ | OR (95% CI)^a^ | p | N (%)^≠^ | OR (95% CI)^a^ | p |  | OR (95% CI)^a,f^ | p | OR (95% CI)^a,g^ | p | p (interaction) |
|  |  |  |  |  |  |  |  |  |  |  |  |  |  |
| Age |  |  | 1.01 (0.99, 1.02) | 0.102 |  | **1.04 (1.03, 1.05)** | **<0.001** |  | 0.99 (0.97, 1.01) | 0.527 | **1.02 (1.00, 1.03)** | **0.019** | 0.576 |
| Sex |  |  |  |  |  |  |  |  |  |  |  |  |  |
| Male |  | 14 (3) | 1 |  | 44 (3) | **1** |  |  | 1 |  | 1 |  |  |
| Female |  | 31 (4) | 1.48 (0.78, 2.80) | 0.214 | 60 (4) | 1.14 (0.61, 2.15) | 0.668 |  | 1.31 (0.71, 2.40) | 0.367 | 1.42 (0.69, 2.88) | 0.320 | 0.786 |
| Older siblings (Yes/No) |  |  |  |  |  |  |  |  |  |  |  |  |  |
| No |  | 9 (2) | 1 |  | 30 (6) | 1 |  |  | 1 |  | 1 |  |  |
| Yes |  | 36 (4) | 1.63 (0.88, 2.96) | 0.109 | 69 (4) | **0.69 (0.52, 0.93)** | **0.016** |  | 1.72 (0.73, 4.08) | 0.204 | **0.63 (0.46, 0.86)** | **0.005** | 0.098 |
| Younger siblings (Yes/No) |  |  |  |  |  |  |  |  |  |  |  |  |  |
| No |  | 7 (2) | 1 |  | 8 (2) | 1 |  |  | 1 |  | 1 |  |  |
| Yes |  | 38 (4) | **2.34 (1.20, 4.55)** | **0.015** | 91 (5) | 1.91 (0.69, 5.23) | 0.190 |  | 1.59 (0.80, 3.15) | 0.171 | 2.07 (0.67, 6.41) | 0.195 | 0.859 |
| Occupation |  |  |  |  |  |  |  |  |  |  |  |  |  |
| Student or child (not at school) |  | 13 (2) | 1 |  | 6 (1) | 1 |  |  | 1 |  | 1 |  |  |
| Unemployed or housewife |  | 19 (7) | **3.46 (1.48, 8.12)** |  | 14 (5) | **6.21 (2.01, 19.1)** |  |  | 4.75 (1.26, 17.9) |  | 1.68 (0.23, 12.5) |  |  |
| Agricultural, fishing or lake related |  | 2 (3) | 1.75 (0.35, 8.84) |  | 58 (4) | **8.22 (2.76, 24.5)** |  |  | 2.15 (0.14, 32.1) |  | 2.60 (0.31, 22.1) |  |  |
| Professional or service providers |  | 11 (3) | 1.76 (0.64, 4.83) | 0.059 | 26 (6) | **8.34 (2.70, 25.7)** | **0.007** |  | 3.17 (0.56, 17.8) | 0.107 | 2.33 (0.25, 21.4) | 0.407 | 0.259 |
| Location of birth |  |  |  |  |  |  |  |  |  |  |  |  |  |
| City |  | 3 (6) | 1 |  | 6 (10) | 1 |  |  | 1 |  | 1 |  |  |
| Town |  | 2 (1) | 0.25 (0.03, 2.28) |  | 14 (6) | **0.48 (0.28, 0.81)** |  |  | 0.09 (0.02, 5.55) |  | **0.50 (0.29, 0.85)** |  |  |
| Village |  | 12 (4) | 0.65 (0.22, 1.93) | 0.456 | 75 (4) | **0.30 (0.14, 0.65)** | **0.009** |  | 0.29 (0.03, 3.05) | 0.496 | **0.28 (0.12, 0.64)** | **0.011** | 0.135 |
| Maternal history of allergies |  |  |  |  |  |  |  |  |  |  |  |  |  |
| No |  | 28 (3) | 1 |  | 68 (3) | 1 |  |  | 1 |  | 1 |  |  |
| Yes |  | 11 (9) | **3.48 (1.35, 8.99)** | **0.012** | 18 (3) | 1.07 (0.56, 2.04) | 0.822 |  | **3.56 (1.28, 9.93)** | **0.017** | 1.58 (0.85, 2.96) | 0.126 | 0.676 |
| Paternal history of allergies |  |  |  |  |  |  |  |  |  |  |  |  |  |
| No |  | 32 (3) | 1 |  | 70 (3) | 1 |  |  | 1 |  | 1 |  |  |
| Yes |  | 6 (9) | **3.07 (1.27, 7.39)** | **0.015** | 10 (3) | 0.79 (0.39, 1.58) | 0.499 |  | **3.29 (1.24, 8.76)** | **0.019** | 1.30 (0.61, 2.76) | 0.479 | 0.435 |
| Lake contact |  |  |  |  |  |  |  |  |  |  |  |  |  |
| Never |  | 9 (2) | 1 |  |  |  |  |  | 1 |  |  |  |  |
| Rarely |  | 23 (4) | **2.15 (1.05, 4.44)** |  | 6 (7) | 1 |  |  | 1.35 (0.51, 3.61) |  | 1 |  |  |
| Once a month |  | 4 (3) | 1.58 (0.43, 5.83) |  |  |  |  |  | 0.55 (0.09, 3.18) |  |  |  |  |
| Once a week |  | 9 (7) | **3.95 (1.85, 8.42)** | **0.004** | 11 (5) | 1.01 (0.28, 3.62) |  |  | 2.24 (0.82, 6.07) | 0.364 | 1.29 (0.35, 4.68) |  |  |
| Daily/ almost daily |  |  |  |  | 82 (4) | 0.84 (0.23, 3.03**)** | 0.651 |  |  |  | 1.30 (0.33, 5.17) | 0.807 |  |
| Bathe in water from lake? |  |  |  |  |  |  |  |  |  |  |  |  |  |
| No |  | 40 (3) | 1 |  | 6 (8) | 1 |  |  | 1 |  | 1 |  |  |
| Yes |  | 5 (4) | 1.36 (0.61, 3.05) | 0.425 | 93 (4) | **0.42 (0.22, 0.79)** | **0.010** |  | 0.89 (0.39, 2.07) | 0.794 | **0.45 (0.27, 0.78)** | **0.006** | 0.223 |
| Hand washing after toilet |  |  |  |  |  |  |  |  |  |  |  |  |  |
| No |  | 2 (1) | 1 |  | 22 (3) | 1 |  |  | 1 |  | 1 |  |  |
| Yes |  | 43 (4) | **4.13 (1.06, 15.9)** | **0.041** | 77 (5) | 1.63 (0.85, 3.14) | 0.135 |  | 2.26 (0.68, 7.47) | 0.169 | 1.91 (0.92, 3.94) | 0.078 | 0.212 |
| HIV |  |  |  |  |  |  |  |  |  |  |  |  |  |
| Negative |  | 37 (3) | 1 |  | 67 (3) | 1 |  |  | 1 |  | 1 |  |  |
| Positive |  | 5 (9) | **2.92 (1.22, 6.99)** | **0.019** | 17 (4) | 1.61 (0.80, 3.22) | 0.172 |  | **3.15 (1.18, 8.39)** | **0.023** | 1.17 (0.57, 2.40) | 0.659 | **0.029** |
|  | | | | | | | | | | | | | |
| *Table shows only factors that were associated with clinical allergy-related outcomes in either the urban or the rural survey, before and/or after adjustment for confounding. All other factors that were assessed are listed in the statistical methods section. Significant associations are highlighted in bold. Interaction p values are shown to denote whether tests for interaction, using the adjusted model, showed statistical evidence for urban-rural differences in associations with wheeze / urticarial rash / rhinitis, or not.*  *≠Number (percentage in parenthesis) of individuals with respective allergic disease in each category*  *^a^Odds ratios (ORs) and 95% confidence intervals (CI) adjusted for survey design.*  *^b^ORs adjusted for hand washing before eating, maternal history of allergies, paternal history of allergies, presence of older siblings, age and sex.*  *^c^ORs adjusted for age and sex.*  *^d^ORs adjusted for maternal history of allergies, paternal history of allergies, alcohol use, age and sex.*  *^e^ORs adjusted for HIV infection status, maternal history of allergies, recent malaria treatment, paternal tribe, maternal tribe, age and sex.*  *^f^ORs adjusted for presence of younger siblings, maternal history of allergies, paternal history of allergies, hand washing after toilet use, HIV infection, age and sex.*  *^g^ORs adjusted for presence of older siblings, location of birth, age and sex.*  **Log_10_ (concentration+1) transformation applied before analysis*  *SWA: Schistosoma adult worm antigen; SEA: Schistosoma egg antigen.* | | | | | | | | | | | | | |
